# Cycle-configuration descriptors: a novel graph-theoretic approach to enhancing molecular inference

**DOI:** 10.1186/s13321-025-01042-z

**Published:** 2025-08-18

**Authors:** Bowen Song, Jianshen Zhu, Naveed Ahmed Azam, Kazuya Haraguchi, Liang Zhao, Tatsuya Akutsu

**Affiliations:** 1 Graduate School of Informatics, Kyoto University, Kyoto, 606-8501 Japan; 2 Department of Mathematics, Quaid-i-Azam University, Islamabad, 45320 Pakistan; 3 Graduate School of Advanced Integrated Studies in Human Survivability, Kyoto University, Kyoto, 606-8306 Japan; 4 Bioinformatics Center, Institute for Chemical Research, Kyoto University, Uji, 611-0011 Japan; 5 Department of Information Sciences, Tokyo University of Science, Noda, Chiba, 278-8510 Japan

**Keywords:** Inverse QSAR/QSPR, Molecular inference, Descriptor design, Mixed integer linear programming, Machine learning

## Abstract

Inference of molecules with desired activities/properties is one of the key and challenging issues in cheminformatics and bioinformatics. For that purpose, our research group has recently developed a state-of-the-art framework mol-infer for molecular inference. This framework first constructs a prediction function for a fixed property using machine learning models, which is then simulated by mixed-integer linear programming to infer desired molecules. The accuracy of the framework heavily relies on the representation power of the descriptors. In this study, we highlight a typical class of non-isomorphic chemical graphs with reasonably different property values that cannot be distinguished by the standard “two-layered (2L) model" of mol-infer. To address this distinguishability problem of the 2L model, we propose a novel family of descriptors, named *cycle-configuration (CC)*, which captures the notion of ortho/meta/para patterns that appear in aromatic rings, which was impossible in the framework so far. Extensive computational experiments show that with the new descriptors, we can construct prediction functions with similar or better performance for all 44 tested chemical properties, including 27 regression datasets and 17 classification datasets comparing with our previous studies, confirming the effectiveness of the CC descriptors. For inference, we also provide a system of linear constraints to formulate the CC descriptors as linear constraints. We demonstrate that a chemical graph with up to 50 non-hydrogen vertices can be inferred within a practical time frame.

## Scientific Contribution

This study proposes a new family of descriptors, *cycle-configuration* (*CC*), for the molecular inference framework mol-infer. Computational experiments demonstrate that incorporating CC descriptors into the 2L model (2L+CC model) can improve the performance of a prediction function in many cases. We also provide an MILP formulation that can infer chemical graphs with up to 50 non-hydrogen atoms within a few minutes.

## Introduction

The problem of inferring molecules that are expected to attain desired activities/properties is one of the fundamental challenges in cheminformatics and bioinformatics. This problem is commonly studied under the name of *quantitative structure activity/property relationship* (*QSAR/QSPR*) and *inverse quantitative structure activity/property relationship* (*inverse QSAR/QSPR*). QSAR/QSPR modeling aims to predict chemical activities/property values based on molecular structures [1, 2], while inverse QSAR/QSPR modeling focuses on inferring molecular structures that exhibit specific chemical activities/property values [3–6]. In this study, we focus on inverse QSAR/QSPR modeling of low molecular weight organic compounds, which has applications in drug discovery [5, 7] and material science [8]. With the recent rapid progress of machine learning (ML), there have been developed a lot of inverse QSAR/QSPR models [9, 10], most of which are deep learning-based generative models; e.g., variational autoencoders [11–14], generative adversarial networks [15–17], invertible flow models [18–20], diffusion models [21, 22], and more recently, transformer architectures [23–26].

However, despite the impressive success achieved by these generative approaches, two important aspects–optimality and exactness–are generally not guaranteed [27], where we mean by optimality the preciseness of a solution to attain the desired activities/properties; and by exactness the guarantee of a solution as a valid molecule. Besides, it is challenging to exploit domain knowledge in these deep learning-based methods, which may limit their practical utility in real-world molecular design tasks.

To address this difficulty, our research group has developed a new framework for molecular inference that is based on mixed integer linear programming (MILP) and ML. This framework, which we call mol-infer, achieves optimality and exactness, and enables practitioners to exploit domain knowledge to some extent. Let ${{\mathcal {G}}}$ denote the set of all possible chemical graphs. The process of mol-infer is summarized as follows.

- *Stage 1:* Determine the target chemical property $\pi$ and collect a data set $D_\pi \subseteq {{\mathcal {G}}}$ of chemical graphs such that the observed value $a({{\mathbb {C}}})$ for the chemical property $\pi$ is available for all chemical graphs ${{\mathbb {C}}}\in D_\pi$.
- *Stage 2:* Design a set of descriptors to obtain a feature function $f:{{\mathcal {G}}}\rightarrow {{\mathbb {R}}}^K$ that converts a chemical graph ${{\mathbb {C}}}\in D_\pi$ into a *K*-dimensional real feature vector $f({{\mathbb {C}}})\in {{\mathbb {R}}}^K$, where *K* is the number of descriptors.
- *Stage 3:* Construct a prediction function $\eta :{{\mathbb {R}}}^K\rightarrow {{\mathbb {R}}}$ from the data set $f(D_\pi )\triangleq \{f({{\mathbb {C}}})\mid {{\mathbb {C}}}\in D_\pi \}$ of feature vectors, where $\eta ({{\mathbb {C}}})$ is used to estimate the property value $a({{\mathbb {C}}})$ of a chemical graph ${{\mathbb {C}}}$.
- *Stage 4:* Determine two real numbers ${\underline{y}}^*,{\overline{y}}^*$ $({\underline{y}}^*\le {\overline{y}}^*)$ as lower/upper bounds on the target value and a set $\sigma$ of rules (called a *specification*) on chemical graphs. Let ${{\mathcal {G}}}_\sigma \subseteq {{\mathcal {G}}}$ denote the set of all chemical graphs that satisfy $\sigma$. Formulate the problem of constructing a chemical graph ${{\mathbb {C}}}^\dagger$ as MILP whose constraints include ${{\mathcal {C}}}_1$ and ${{\mathcal {C}}}_2$ to ensure $({{\mathcal {C}}}_1)$ ${\underline{y}}^*\le \eta (f({{\mathbb {C}}}^\dagger ))\le {\overline{y}}^*$ and $({{\mathcal {C}}}_2)$ ${{\mathbb {C}}}^\dagger \in {{\mathcal {G}}}_\sigma$. Solve the MILP to obtain ${{\mathbb {C}}}^\dagger$. If the MILP is infeasible, then it is indicated that no such ${{\mathbb {C}}}^\dagger$ exist.
- *Stage 5:* Generate isomers of ${{\mathbb {C}}}^\dagger$ somehow.

**Fig. 1:**
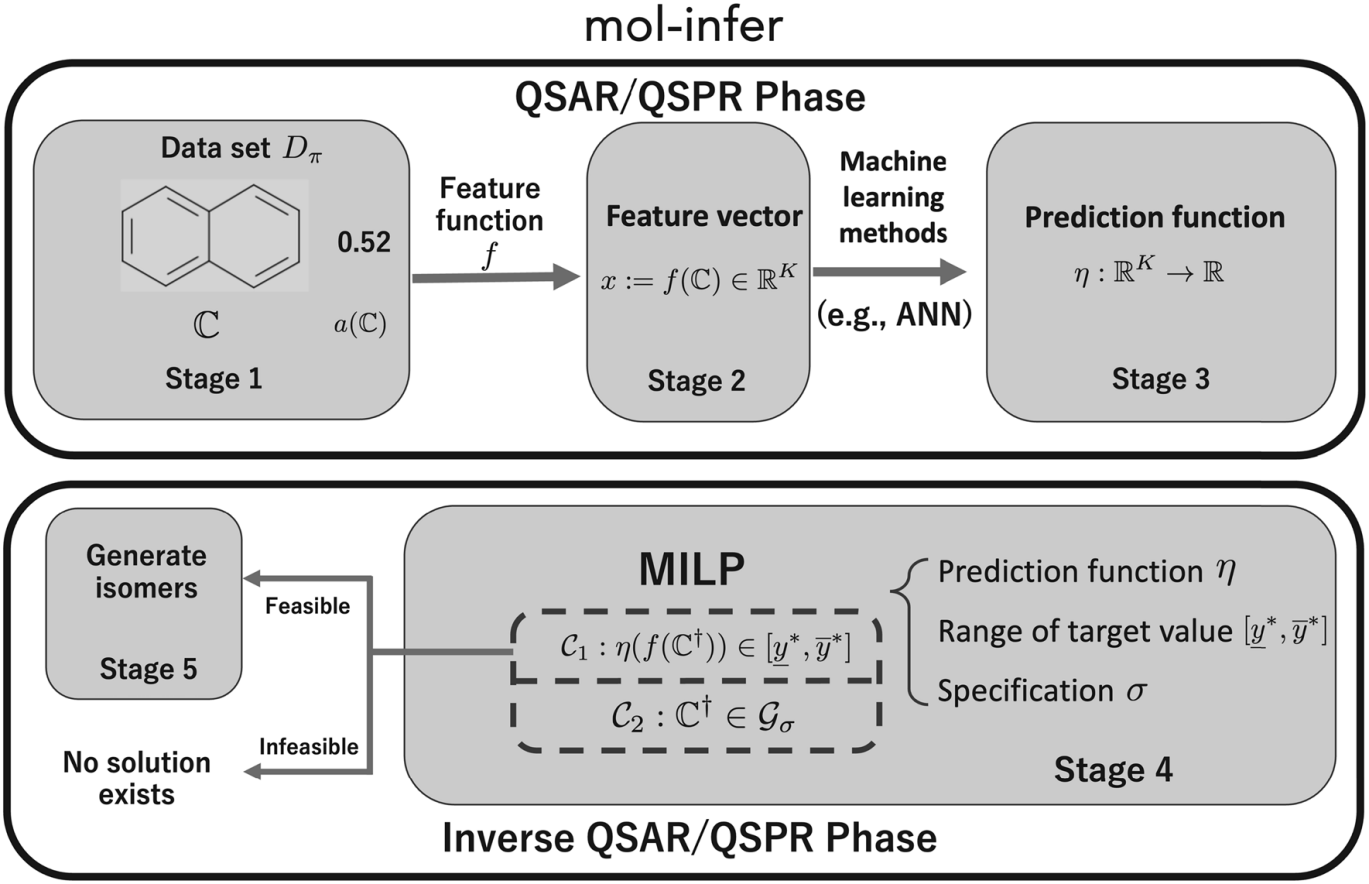
An overview of the mol-infer framework for molecular inference

See Fig. 1 for an illustration of the mol-infer framework.

Regarded as a method of inverse QSAR/QSPR, the highlight of mol-infer is Stage 4 that solves the inverse problem by MILP, which is the original contribution of this framework. For ${{\mathcal {C}}}_1$, the process of computing the feature vector $f({{\mathbb {C}}})$ for a chemical graph ${{\mathbb {C}}}$ and the process of computing the prediction value $\eta (x)$ of a feature vector $x=f({{\mathbb {C}}})$ must be represented by linear inequalities of real and/or integer variables. It is shown that artificial neural network [28], linear regression [29] and decision tree [30] can be used as $\eta$. We will discuss how to design *f* for this purpose in the next paragraph. For ${{\mathcal {C}}}_2$, in our early studies, we could deal with only limited classes of chemical graphs; e.g., trees [31, 32], rank-1 graphs [33] and rank-2 graphs [34]. Shi et al.’s *two-layered* (*2L*) *model* [35] admits us to infer *any* chemical graph, where users are required to design an abstract structure of ${{\mathbb {C}}}^\dagger$ as a part of the specification $\sigma$. Stage 5 is not within the scope of this paper, thus the detail is omitted. Furthermore, mol-infer is applied to the inference of polymers [36].

Let us describe how we design the feature function *f* in Stage 2. The descriptors should be informative since they have a great influence on prediction performance in Stage 3 and thus on the quality of chemical graphs that we finally obtain as a result of Stages 4 and 5; if the prediction function $\eta$ is not accurate enough, then we could not expect the inferred graphs to have desired property. On the other hand, as mentioned above, the process of computing descriptor values should be represented by a set of linear inequalities. It is hard to include descriptors of complicated concepts. There is a trade-off between informativity and simplicity in the design of descriptors.

Due to these reasons, mol-infer employs graph-theoretic descriptors that capture local information of chemical graphs and that are somewhat similar to typical fingerprints. Let $f_{\text {2L}}$ be a feature function in the 2L model, the standard model in mol-infer. The 2L model has a weak point that there are distinct chemical graphs ${{\mathbb {C}}}_1,{{\mathbb {C}}}_2$ for which $f_{\text {2L}}({{\mathbb {C}}}_1)=f_{\text {2L}}({{\mathbb {C}}}_2)$ holds but $a({{\mathbb {C}}}_1)$ and $a({{\mathbb {C}}}_2)$ are much different. See Sect. ''Motivation'' for the details. This issue comes from that the descriptors of the 2L model cannot capture how edges are connected to cycles. Such a phenomenon can be problematic because it will substantially degrade the predictive performance of the constructed prediction function. As a result, the quality of the chemical graphs generated in Stage 4 is also adversely affected, since it depends heavily on the accuracy of the prediction function built in Stage 3. Thus, addressing this weak point is very important for improving the overall performance and reliability of our framework.

In this study, aiming at overcoming the above weak point in the 2L model, we propose a novel set of descriptors, named *cycle-configurations* (*CC*). CC can specify how exterior parts (called “fringe-trees”) are attached to a cycle, by which meta/para patterns in an aromatic ring are distinguishable. Let us denote by $f_{\text {CC}}$ a feature function that consists of CC descriptors.

We call the 2L model with CC descriptors the *2L*$+$*CC model*. In the 2L$+$CC model, we use the feature function $f:{{\mathcal {G}}}\rightarrow {{\mathbb {R}}}^K$ such that $f({{\mathbb {C}}}):=(f_{\text {2L}}({{\mathbb {C}}}),f_{\text {CC}}({{\mathbb {C}}}))$ for a chemical graph ${{\mathbb {C}}}$ (i.e., concatenation of two feature vectors $f_{\text {2L}}({{\mathbb {C}}})$ and $f_{\text {CC}}({{\mathbb {C}}})$).

Computational experiments show that, by using the 2L$+$CC model, we can construct prediction functions of similar or better performance for all of the $27+17=44$ tested chemical properties, in comparison with the 2L model, where 27 data sets are for regression and 17 data sets are for binary classification. We also provide an MILP formulation for the 2L$+$CC model that asks for a chemical graph with desired properties. We show that a chemical graph with up to 50 non-hydrogen vertices can be inferred in a couple of minutes. This is considered the state-of-the-art result.

The paper is organized as follows. We make preparations and review the 2L model in Sect. “Preliminaries''. In Sect. “Cycle-Configurations'', we describe further background of CC and provide its formal definition. In Sect. “MILP Formulation for 2L+CC Model'', we describe the idea of the MILP for the 2L+CC model. We present computational results in Sect. “Results and Discussion'' and conclude the paper in Sect. “Conclusions''. Some details are explained in Appendix. A preprint version of this work appeared in arXiv [37], and a preliminary version of this work was presented as a short paper in the proceedings of the 2024 IEEE International Conference on Bioinformatics and Biomedicine (BIBM) [38]. This work substantially extends the previous work by including additional experimental results on classification data sets, more results on the inference tasks, and a detailed MILP formulation for molecular inference, none of which were presented in the original short paper.

## Preliminaries

### Notations and terminologies

Let ${{\mathbb {R}}}$, ${{\mathbb {R}}}_+$, ${{\mathbb {Z}}}$ and ${{\mathbb {Z}}}_+$ denote the sets of reals, non-negative reals, integers, and non-negative integers, respectively. For $p,q\in {{\mathbb {Z}}}$, let us denote $[p,q]:=\{p,p+1,\dots ,q\}$. For a vector (or a sequence) $x\in {{\mathbb {R}}}^p$ and $j\in [1,p]$, we denote by *x*(*j*) the *j*-th entry of *x*. We denote $|x|:=p$.

Let *A* be a finite set. To encode elements in *A* by integers, we may assume a bijection $\sigma :A\rightarrow [1,|A|]$ implicitly. For $a\in A$, we represent the coded integer $\sigma (a)$ by $[a]_A$ or simply [*a*] if *A* is clear from the context.

For an undirected graph *G*, we denote by *V*(*G*) and *E*(*G*) the sets of vertices and edges, respectively. For $V'\subseteq V(G)$ (resp., $E'\subseteq E(G)$), we denote by $G-V'$ (resp., $G-E'$) the subgraph of *G* that is obtained by removing the vertices in $V'$ along with the incident edges (resp., removing the edges in $E'$). When $V'=\{v\}$ (resp., $E'=\{e\}$), we write $G-\{v\}$ as $G-v$ (resp., $G-\{e\}$ as $G-e$).

A *cycle* *C* in a graph *G* is a subgraph of *G* such that $V(C)=\{u_1,u_2,\dots ,u_{\ell }\}$ and $E(C)=\{u_1u_2,\dots ,u_{\ell -1}u_{\ell },u_{\ell }u_1\}$. We call *C* *chordless* if there is no edge in $E(G)\setminus E(C)$ that joins vertices in *V*(*C*). The length of a cycle *C* is denoted by ${\rm len}(C)$ (i.e., ${\rm len}(C)=|V(C)|=|E(C)|=\ell$). When the length is $\ell$, we call *C* an $\ell$-*cycle*.

A graph is *rooted* if it has a designated vertex, called a *root*. For a graph *G* possibly with a root, a *leaf-vertex* is a non-root vertex with degree 1. We call the edge that is incident to a leaf-vertex a *leaf-edge*. We denote by $V_{\text {leaf}}(G)$ and $E_{\text {leaf}}(G)$ the sets of leaf-vertices and leaf-edges in *G*, respectively. For $i\in {{\mathbb {Z}}}_+$, we define the graph $G_i$ to be the subgraph of *G* that is obtained by deleting the set of leaf-vertices *i* times, that is, $G_0:=G$; and $G_{i+1}:=G_i-V_{\text {leaf}}(G_i)$. We define the *height* ${\rm ht}(v)$ of a vertex $v\in V_{\text {leaf}}(G_i)$ to be *i*. Note that the height may not be defined for all vertices.

### Modeling of chemical compounds

We employ the modeling of chemical compounds that was introduced by Zhu et al. [29].

Let us represent chemical elements by H (hydrogen), C (carbon), O (oxygen), N (nitrogen) and so on. To distinguish a chemical element a with multiple valences such as S (sulfur), we denote a with a valence *i* by a$_{(i)}$, where we omit the suffix (*i*) for a chemical element with a unique valence. Let $\Lambda$ be a set of chemical elements; e.g., $\Lambda =\{{\mathtt H},{\mathtt C},{\mathtt O},{\mathtt N},{\mathtt P},{\mathtt S}_{(2)},{\mathtt S}_{(4)},{\mathtt S}_{(6)}\}$. We represent the valence of ${\mathtt a}\in \Lambda$ by a function $\rm{val}:\Lambda \rightarrow [1,6]$; e.g., $\rm{val}({\mathtt H})=1$, $\rm{val}({\mathtt C})=4$, $\rm{val}({\mathtt O})=2$, $\rm{val}({\mathtt P})=5$, $\rm{val}({\mathtt S}_{(2)})=2$ and $\rm{val}({\mathtt S}_{(6)})=6$. We denote the mass of ${\mathtt a}\in \Lambda$ by $\rm{mass}^*({\mathtt a})$.

We represent a chemical compound by a *chemical graph* that is defined to be ${{\mathbb {C}}}=(H,\alpha ,\beta )$ consisting of a simple, connected undirected graph *H* and functions $\alpha :V(H)\rightarrow \Lambda$ and $\beta :E(H)\rightarrow [1,3]$. The set of atoms and the set of bonds in the compound correspond to the vertex set *V*(*H*) and the edge set *E*(*H*), respectively. The chemical element assigned to $v\in V(H)$ is represented by $\alpha (v)$ and the bond-multiplicity between two adjacent vertices $u,v\in V(H)$ is represented by $\beta (e)$ of the edge $e=uv\in E(H)$. We denote the mass of *H* by ${\rm mass}^*(H):=\sum _{v\in V(H)}{\rm mass}^*(\alpha (v))$.

Let ${{\mathbb {C}}}=(H,\alpha ,\beta )$ be a chemical graph. For a vertex $u\in V(H)$, we denote by $\beta _{{\mathbb {C}}}(u)$ the sum of bond-multiplicities of edges incident to *u*; i.e., $\displaystyle \beta _{{\mathbb {C}}}(u):=\sum _{uv\in E(H)}\beta (uv)$. We denote by $\deg _{{\mathbb {C}}}(u)$ the number of vertices adjacent to *u* in ${{\mathbb {C}}}$. For ${\mathtt a}\in \Lambda$, we denote by $V_{{\mathtt a}}({{\mathbb {C}}})$ the set of vertices in $v\in V(H)$ such that $\alpha (v)={\mathtt a}$ in ${{\mathbb {C}}}$. We define the *hydrogen-suppressed chemical graph of* ${{\mathbb {C}}}$, denoted by $\langle {{\mathbb {C}}}\rangle$, to be the graph that is obtained by removing all vertices in $V_{{\mathtt H}}({{\mathbb {C}}})$ from *H*.

Two chemical graphs ${{\mathbb {C}}}_i=(H_i,\alpha _i,\beta _i)$, $i=1,2$ are called *isomorphic* if they admit an isomorphism, i.e., a bijection $\phi :V(H_1)\rightarrow V(H_2)$ such that “$uv\in E(H_1)$, $\alpha _1(u)={\mathtt a}$, $\alpha _1(v)={\mathtt b}$, $\beta _1(uv)=m$” $\Leftrightarrow$ “$\phi (u)\phi (v)\in E(H_2)$, $\alpha _2(\phi (u))={\mathtt a}$, $\alpha _2(\phi (v))={\mathtt b}$, $\beta _2(\phi (u)\phi (v))=m$”. Furthermore, when $H_i$ is a rooted graph such that $r_i\in V(H_i)$ is the root, $i=1,2$, ${{\mathbb {C}}}_1$ and ${{\mathbb {C}}}_2$ are called *rooted-isomorphic* if they admit an isomorphism such that $\phi (r_1)=r_2$.

### Two-layered (2L) model

We review the 2L model that was introduced by Shi et al. [35] for completeness.

#### Interior and exterior

Let ${{\mathbb {C}}}=(H,\alpha ,\beta )$ be a chemical graph and $\rho \ge 1$ be an integer, which we call a *branch-parameter*, where we use $\rho =2$ as the standard value. In the 2L model, the hydrogen-suppressed chemical graph $\langle {{\mathbb {C}}}\rangle$ is partitioned into “interior” and “exterior” parts as follows. We call a vertex $v\in V(\langle {{\mathbb {C}}}\rangle )$ an *exterior-vertex* if ${\rm ht}(v)<\rho$, and an edge $e\in E(\langle {{\mathbb {C}}}\rangle )$ an *exterior-edge* if *e* is incident to an exterior-vertex. Let $V^{\text {ex}}({{\mathbb {C}}})$ and $E^{\text {ex}}({{\mathbb {C}}})$ denote the sets of exterior-vertices and exterior-edges, respectively. Define $V^{\text {int}}({{\mathbb {C}}}):=V(\langle {{\mathbb {C}}}\rangle )\setminus V^{\text {ex}}({{\mathbb {C}}})$ and $E^{\text {int}}({{\mathbb {C}}}):=E(\langle {{\mathbb {C}}}\rangle )\setminus E^{\text {ex}}({{\mathbb {C}}})$. We call a vertex in $V^{\text {int}}({{\mathbb {C}}})$ an *interior-vertex* and an edge in $E^{\text {int}}({{\mathbb {C}}})$ an *interior-edge*. We define the *interior* ${{\mathbb {C}}}^{\text {int}}$ *of* ${{\mathbb {C}}}$ to be the subgraph $(V^{\text {int}}({{\mathbb {C}}}),E^{\text {int}}({{\mathbb {C}}}))$.

The set $E^{\text {ex}}({{\mathbb {C}}})$ of exterior-edges forms a collection of connected graphs such that each is a tree *T* rooted at an interior vertex $v\in V(T)$. Let ${{\mathcal {T}}}^{\text {ex}}(\langle {{\mathbb {C}}}\rangle )$ denote the family of such chemical rooted trees in $\langle {{\mathbb {C}}}\rangle$. For each interior-vertex $u\in V^{\text {int}}({{\mathbb {C}}})$, let $T_u\in {{\mathcal {T}}}^{\text {ex}}(\langle {{\mathbb {C}}}\rangle )$ denote the chemical tree rooted at *u*, where $T_u$ may consist only of the vertex *u*. We define the *fringe-tree of* *u*, denoted by ${{\mathbb {C}}}[u]$, to be the chemical rooted tree that is obtained by putting back hydrogens to $T_u$ that are originally attached in ${{\mathbb {C}}}$.

#### Feature function

For a feature function $f_{\text {2L}}$ in the 2L model (Stage 2), there are two types of descriptors: static ones and enumerative ones. There are 14 static descriptors such as the number of non-hydrogen atoms and the number of interior vertices. The enumerative descriptors mainly consist of the frequency of local patterns that appear in a chemical graph ${{\mathbb {C}}}=(H,\alpha ,\beta )$. Examples of such local patterns include “fringe-configurations”, “adjacency-configurations” and “edge-configurations”. We collect enumerative descriptors from a given data set $D_\pi$.

Let $u\in V^{\text {int}}({{\mathbb {C}}})$ be an interior-vertex. The *fringe-configuration of u* is the chemical tree ${{\mathbb {C}}}[u]$ that is rooted at *u*. Let us denote by ${{\mathcal {F}}}(D_\pi )$ the set of all fringe trees that appear in the data set $D_\pi$. For each $\psi \in {{\mathcal {F}}}(D_\pi )$, we introduce a descriptor that evaluates the number of interior-vertices $u\in V^{\text {int}}({{\mathbb {C}}})$ such that ${{\mathbb {C}}}[u]$ is rooted-isomorphic to $\psi$.

For an interior-edge $e=uv\in E^{\text {int}}({{\mathbb {C}}})$, let $\alpha (u)={\mathtt a}$, $\deg _{\langle {{\mathbb {C}}}\rangle }(u)=d$, $\alpha (v)={\mathtt b}$, $\deg _{\langle {{\mathbb {C}}}\rangle }(v)=d'$ and $\beta (e)=m$. The *adjacency-configuration of e* (resp., *edge-configuration of* *e*) is defined to be the tuple $({\mathtt a},{\mathtt b},m)$ (resp., $({\mathtt a}d,{\mathtt b}d',m)$). Let us denote by $\Gamma _{\text {ac}}(D_\pi )$ (resp., $\Gamma _{\text {ec}}(D_\pi )$) the set of all adjacency-configurations (resp., edge-configurations) in the data set $D_\pi$. For each tuple $\gamma _{\text {ac}}\in \Gamma _{\text {ac}}(D_\pi )$ (resp., $\gamma _{\text {ec}}\in \Gamma _{\text {ec}}(D_\pi )$), we introduce a descriptor that evaluates the number of interior-edges $e\in E^{\text {int}}({{\mathbb {C}}})$ such that the adjacency-configuration (resp., edge-configuration) is equal to $\gamma _{\text {ac}}$ (resp., $\gamma _{\text {ec}}$).

See Appendix A for a full description of descriptors in the 2L model.

#### Specification for MILP

In the 2 L model, the specification $\sigma$ for MILP (Stage 4) consists of the following three rules:

- A *seed graph* $G_{\text {C}}$ as an abstract form of a target chemical graph ${{\mathbb {C}}}^\dagger$;
- A set ${{\mathcal {F}}}$ of fringe trees as candidates for a tree ${{\mathbb {C}}}^\dagger [u]$ rooted at each interior-vertex *u* in ${{\mathbb {C}}}^\dagger$; and
- Lower/upper bounds on the number of various parameters in ${{\mathbb {C}}}^\dagger$; e.g., chemical elements, double/triple bonds, and fringe/edge/adjacency-configurations.

The MILP formulates the process of constructing a chemical graph ${{\mathbb {C}}}^\dagger$ as follows. First, we decide the interior of ${{\mathbb {C}}}^\dagger$ by “expanding” the seed graph $G_{\text {C}}$; e.g., subdividing an edge and attaching a new path to a vertex. Second, regarding all vertices in the expanded seed graph as the interior-vertices of ${{\mathbb {C}}}^\dagger$, we assign a fringe tree in ${{\mathcal {F}}}$ to every vertex to make the exterior of ${{\mathbb {C}}}^\dagger$. Finally, we assign bond-multiplicities to the interior-edges so that all constraints in $\sigma$ are satisfied. We can regard ${{\mathcal {G}}}_\sigma$ in Sect. ''Introduction'' as the set of all chemical graphs that can be constructed in this way. See the preprint of [29] for details of MILP in the 2L model.

## Cycle-configurations

In this section, we propose a new type of descriptors for the 2L model, named cycle-configurations (CC). 

### Motivation

**Fig. 2:**
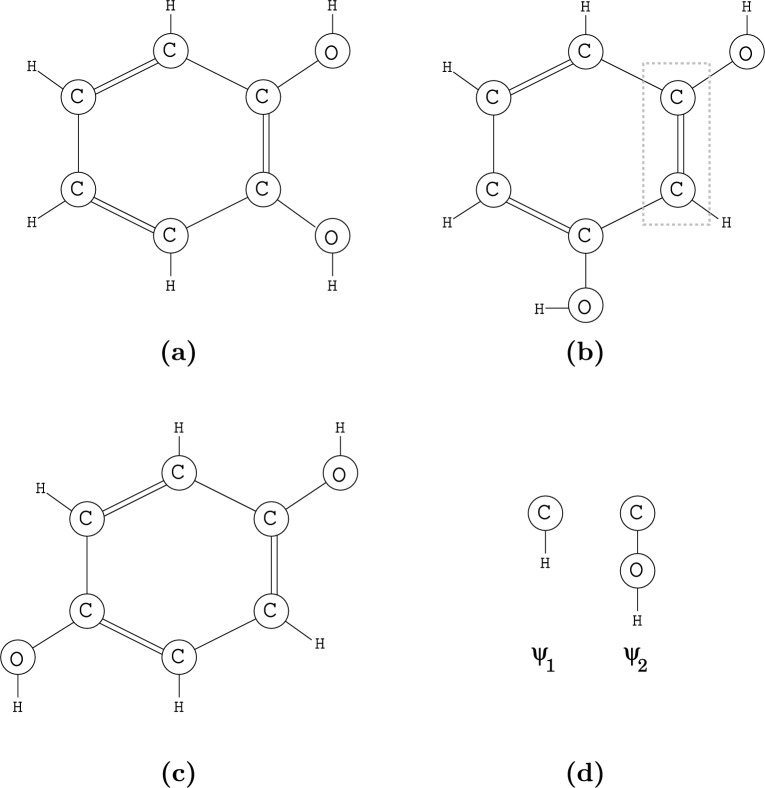
**a** the chemical graph ${{\mathbb {C}}}_0$ for catechol; **b** the chemical graph ${{\mathbb {C}}}_1$ for resorcinol; **c** the chemical graph ${{\mathbb {C}}}_2$ for hydroquinone; and **d** two fringe-trees $\psi _1$ and $\psi _2$ that appearing in all of ${{\mathbb {C}}}_0$, ${{\mathbb {C}}}_1$ and ${{\mathbb {C}}}_2$. In **b**, the edge-configuration of the interior-edge indicated by a dotted rectangle is $({\mathtt C}2,{\mathtt C}3,2)$. Although $f_{\text {2L}}({{\mathbb {C}}}_1)=f_{\text {2L}}({{\mathbb {C}}}_2)$, $a({{\mathbb {C}}}_1)=0\ne 1=a({{\mathbb {C}}}_2)$ holds in the data set of AhR property from Tox21 collection

Let us point out a weak point of the 2L model precisely. There are chemical graphs that are not isomorphic to each other but are converted into an identical feature vector. See Fig. 2 for an example. Three chemical graphs ${{\mathbb {C}}}_0$ (catechol), ${{\mathbb {C}}}_1$ (resorcinol) and ${{\mathbb {C}}}_2$ (hydroquinone) are shown, where ${{\mathbb {C}}}_0$ is the ortho-isomer, ${{\mathbb {C}}}_1$ is the meta-isomer and ${{\mathbb {C}}}_2$ is the para-isomer.

We can confirm that $f_{\text {2L}}({{\mathbb {C}}}_1)=f_{\text {2L}}({{\mathbb {C}}}_2)$ holds by observing the descriptors one by one. For fringe-configuration, both chemical graphs contain four $\psi _1$ and two $\psi _2$ as fringe-trees in common. For edge-configuration, they contain one $({\mathtt C}2,{\mathtt C}2,1)$; one $({\mathtt C}2,{\mathtt C}2,2)$; two $({\mathtt C}2,{\mathtt C}3,1)$ and two $({\mathtt C}2,{\mathtt C}3,2)$ in common. In this way, one sees that the two chemical graphs take the same values for the other descriptors (see Appendix A). We also see that $f_{\text {2L}}({{\mathbb {C}}}_0)\ne f_{\text {2L}}({{\mathbb {C}}}_1)$ and $f_{\text {2L}}({{\mathbb {C}}}_0)\ne f_{\text {2L}}({{\mathbb {C}}}_2)$ hold since ${{\mathbb {C}}}_0$ contains one $({\mathtt C}2,{\mathtt C}2,1)$; two $({\mathtt C}2,{\mathtt C}2,2)$; two $({\mathtt C}2,{\mathtt C}3,1)$ and one $({\mathtt C}3,{\mathtt C}3,2)$ for its edge-configurations, which are different from those of ${{\mathbb {C}}}_1$ and ${{\mathbb {C}}}_2$. In summary, although the descriptors of the 2L model can distinguish ortho patterns of an aromatic ring from meta/para patterns, they fail to distinguish meta and para patterns.

Although the two chemical graphs ${{\mathbb {C}}}_1$ and ${{\mathbb {C}}}_2$ are converted into an identical feature vector, they may have different properties from each other. For example, Tox21 [39] is a collection of data sets for binary classification (i.e., $a({{\mathbb {C}}})\in \{0,1\}$ for ${{\mathbb {C}}}\in D_\pi$). In AhR data set, the two chemical graphs ${{\mathbb {C}}}_1$ and ${{\mathbb {C}}}_2$ in Fig. 2 satisfy $f_{\text {2L}}({{\mathbb {C}}}_1)=f_{\text {2L}}({{\mathbb {C}}}_2)$ although $a({{\mathbb {C}}}_1)=0$ and $a({{\mathbb {C}}}_2)=1$ hold. It is desirable to convert as many such pairs into distinct feature vectors as possible.

### Definitions

We define a new descriptor, cycle-configuration, in order to convert chemical graphs like ${{\mathbb {C}}}_1$ and ${{\mathbb {C}}}_2$ in Fig. 2 into distinct feature vectors. Let $R=\{a_1,a_2,\dots ,a_k\}$ be a set of distinct real numbers. For $a\in R$, we define ${\rm rank}_R(a):=i$ if *a* is the *i*-th smallest in *R*. For example, when $R=\{3,2,5,9\}$, we have ${\rm{rank}}_R(3)=2$, ${\rm{rank}}_R(2)=1$, ${\rm{rank}}_R(5)=3$, and ${\rm{rank}}_R(9)=4$.

Suppose that a chemical graph ${{\mathbb {C}}}$ is given. Let *C* be a chordless cycle in ${{\mathbb {C}}}$ such that $V(C)=\{u_1,u_2,\dots ,u_{\ell }\}$ and $E(C)=\{u_1u_2,u_2u_3,\dots ,u_{\ell -1}u_{\ell },u_{\ell }u_1\}$. For $u_i\in V(C)$, we define $\mu _i:={\rm mass}^*({{\mathbb {C}}}[u_i])$. Let *R* denote the set of distinct numbers in $\mu _1,\mu _2,\dots ,\mu _\ell$. We define $\xi (C)$ to be the smallest sequence $({\rm rank}_R(\mu _1),{\rm rank}_R(\mu _2),\dots ,{\rm rank}_R(\mu _{\ell }))$ with respect to the lexicographic order among all possible cyclic permutations (including reversal) of $(u_1,u_2,\dots ,u_{\ell })$, where there are $2\ell$ permutations possible. We define the *cycle-configuration of C* to be $\xi (C)$.

Let us see Fig. 2 for example. Suppose $\rm{mass}^*({\mathtt H})=1$, $\rm{mass}^*({\mathtt C})=12$ and $\rm{mass}^*({\mathtt O})=16$. For the two fringe-trees $\psi _1$ and $\psi _2$ in the figure, we have $\rm{mass}^*(\psi _1)=12+1=13$ and $\rm{mass}^*(\psi _2)=12+16+1=29$. Let us denote the unique (chordless) 6-cycle in ${{\mathbb {C}}}_i$ by $C_i$, $i=1,2$. The set of distinct numbers that appear as the mass of a fringe-tree is $R=\{13,29\}$ for both chordless cycles, where ${\rm rank}_R(13)=1$ and ${\rm rank}_R(29)=2$. One readily sees that $\xi (C_1)=\xi _1:=(1,1,1,2,1,2)$ and $\xi (C_2)=\xi _2:=(1,1,2,1,1,2)$.

CCs are enumerative descriptors, and we collect ones that are included in the feature function from a given data set $D_\pi$. We denote by $\Xi (D_\pi )$ the set of all cycle-configurations $\xi$ that appear in $D_\pi$. Let $K_{\text {CC}}:=|\Xi (D_\pi )|$. For a chemical graph ${{\mathbb {C}}}\in D_\pi$, we define $f_{\text {CC}}({{\mathbb {C}}})$ to be a $K_{\text {CC}}$-dimensional feature vector $f_{\text {CC}}({{\mathbb {C}}})=({\rm dcp}^\circ _1({{\mathbb {C}}}),{\rm dcp}^\circ _2({{\mathbb {C}}}),\dots ,{\rm dcp}^\circ _{K_{\text {CC}}}({{\mathbb {C}}}))$, where

${\rm dcp}^\circ _i({{\mathbb {C}}})$, $i=[\xi ^*]$, $\xi ^*\in \Xi (D_\pi )$: the number of chordless cycles *C* in ${{\mathbb {C}}}$ such that $\xi (C)=\xi ^*$.

See Fig. 2 again. Suppose $\Xi (D_\pi )=\{\xi _1,\xi _2,\xi _3,\xi _4\}$ for $\xi _3:=(1,1,2,3)$ and $\xi _4:=(1,1,1,1,2)$. Then $f_{\text {CC}}({{\mathbb {C}}}_1)=(1,0,0,0)$ and $f_{\text {CC}}({{\mathbb {C}}}_2)=(0,1,0,0)$ hold, by which we have $f({{\mathbb {C}}}_1)=(f_{\text {2L}}({{\mathbb {C}}}_1),f_{\text {CC}}({{\mathbb {C}}}_1))\ne (f_{\text {2L}}({{\mathbb {C}}}_2),f_{\text {CC}}({{\mathbb {C}}}_2))=f({{\mathbb {C}}}_2)$.

In our implementation, as $D_\pi$ may contain too many CC descriptors, we use only CC descriptors whose lengths are in the range $[c_{\min },c_{\max }]$, where $c_{\min }$ and $c_{\max }$ are positive constants $(c_{\min }\le c_{\max })$. We will set $c_{\min }:=4$ and $c_{\max }:=6$ since, in most of chemical compounds in conventional databases, the chemical graph is acyclic or contain only chordless cycles whose lengths are within [4, 6]. See Table 1. For example, in PubChem, among 92,509,596 molecules that are feasible in the 2L-model, 83,520,760 molecules (90%) satisfy this condition.

**Table 1:** The numbers of chemical compounds in conventional databases. The 2nd to 5th columns represent the number of all registered chemical compounds; the number of feasible chemical graphs in the 2L-model (e.g., connected, at least four carbon atoms exist); the number of chemical graphs that are either acyclic or $\ell (C)\in [4,6]$ for all chordless cycles *C*; the number of chemical graphs that contain none of (i) or (ii) in Sect. ''MILP Formulation for 2L+CC Model'', respectively. The percentages indicate the ratio of the number over the left number

Database	All	2L-model	Acyclic or $\ell (C)\in [4,6]$	No substructures
		feasible	for all chordless cycles *C*	(i) or (ii) in Section ''MILP Formulation for 2L+CC Model''
PubChem	97,092,888	92,509,596	83,520,760	80,842,345
(as of 2019)		(95%)	(90%)	(96%)
QM9	130,786	130,786	71,520	60,352
		(100%)	(54%)	(84%)
Tox21	8,014	7,769	7,273	7,080
		(96%)	(93%)	(97%)

## MILP formulation for 2L$+$CC Model

Let us consider an MILP formulation for inferring a chemical graph in the 2L$+$CC model. Similarly to the 2L model, the constraints of the MILP consist of $({{\mathcal {C}}}_1)$ ${\underline{y}}^*\le \eta (f({{\mathbb {C}}}^\dagger ))\le {\overline{y}}^*$ and $({{\mathcal {C}}}_2)$ ${{\mathbb {C}}}^\dagger \in {{\mathcal {G}}}_\sigma$, where ${{\mathbb {C}}}^\dagger$ denotes a chemical graph to be inferred and is represented by real/integer variables. We can use any prediction function $\eta$ in ${{\mathcal {C}}}_1$ if its computational process can be represented by a set of linear inequalities. For example, artificial neural network [28], linear regression [29] and decision tree [30] can be used to construct $\eta$. In this section, we overview how we formulate ${{\mathcal {C}}}_2$ as MILP. See Appendix B for the precise formulation of the MILP that includes how we represent the computational process of the feature function *f* by a set of linear inequalities.

The basic idea of ${{\mathcal {C}}}_2$ is similar to the 2L model (see Sect. ''Specification for MILP''); we represent by ${{\mathcal {C}}}_2$ the computational process of expanding an abstract form of the chemical graph to a concrete chemical graph. We introduce a new type of abstract form, which we call a “seed tree”, since it is hard to deal with CC descriptors by a seed graph of the 2L model.

**Fig. 3:**
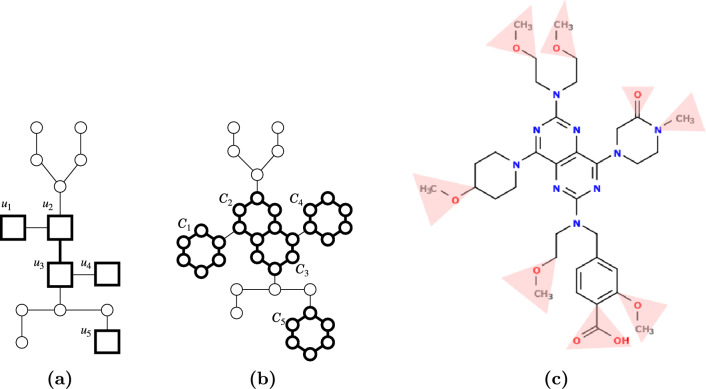
Construction of a chemical graph. **a** A seed tree. Thick squares/lines indicate ring nodes/edges, while thin circles/lines indicate non-ring nodes/edges. **b** Ring nodes are expanded to chordless 6-cycles. **c** Fringe-trees are assigned to every vertex and bond-multiplicities are assigned to every edge. Fringe-trees of non-zero heights are indicated by shade. The PubChem CID of the compound is 156,839,899, and the molecular formula is $\hbox {C}_{35} \hbox {H}_{51} \hbox {N}_9 \hbox {O}_8$

A *seed tree* is a tuple ${{\mathcal {T}}}=(T;V^{\circ },E^{\circ })$ of an unrooted tree *T*, $V^{\circ }\subseteq V(T)$ and $E^{\circ }\subseteq \{uv\in E(T)\mid u,v\in V^{\circ }\}$. We call a node in $V^{\circ }$ a *ring node* and an edge in $E^{\circ }$ a *ring edge*, whereas a node in $V(T)\setminus V^{\circ }$ is a *non-ring node*, and an edge in $E(T)\setminus E^{\circ }$ is a *non-ring edge*. For a node $u\in V(T)$, we denote by $E^{\circ }(u)$ and ${\bar{E}}^{\circ }(u)$ the sets of all ring edges and of all non-ring edges incident to *u*, respectively. A demonstration of obtaining a specific molecule from a seed tree is given in Fig. 3.

We formulate by ${{\mathcal {C}}}_2$ the following process of constructing a chemical graph ${{\mathbb {C}}}^\dagger :={{\mathbb {C}}}_{{{\mathcal {T}}}}=(G_{{{\mathcal {T}}}},\alpha ,\beta )$.

(I) Each ring node $u\in V^{\circ }$ is assigned a cycle-configuration, by which *u* is “expanded” to a chordless cycle in $G_{{{\mathcal {T}}}}$.
  If two ring nodes are joined by a ring edge, then the corresponding two chordless cycles in $G_{{{\mathcal {T}}}}$ share an edge in common.
  Each non-ring node in $V(T)\setminus V^{\circ }$ appears as a single vertex in $G_{{{\mathcal {T}}}}$.
  Each non-ring edge in $E(T)\setminus E^{\circ }$ appears as a single edge in $G_{{{\mathcal {T}}}}$.
(II) The expanded graph is used as the interior of $G_{{{\mathcal {T}}}}$. For the exterior, fringe-trees are assigned to all nodes in the expanded graph, and to the interior-edges, bond-multiplicities are assigned.

For the seed tree ${{\mathcal {T}}}=(T;V^{\circ },E^{\circ })$ in Fig. 3(a), we have $V^{\circ }=\{u_1,u_2,\dots ,u_5\}$. Suppose that we are given $\Xi ^u=\{\xi _1,\xi _2,\dots ,\xi _6\}$ for all ring nodes $u\in V^{\circ }$, where

$$\begin{aligned}&\xi _1=(1,1,2,3), & \xi _2=(1,1,1,1,2), & \xi _3=(1,1,1,1,1,2),\\&\xi _4=(1,1,1,1,2,3), & \xi _5=(1,1,1,2,1,2), & \xi _6=(1,2,2,4,3,2). \end{aligned}$$

In this example, $u_1$ is assigned $\xi _3$; $u_2$ and $u_3$ are assigned $\xi _5$; $u_4$ is assigned $\xi _4$; and $u_5$ is assigned $\xi _6$, where $\xi _1$ and $\xi _2$ are assigned to no ring nodes. As shown in Fig. 3(b), all ring nodes are expanded to chordless 6-cycles, $C_1$ to $C_5$, where $6=|\xi _3|=|\xi _4|=|\xi _5|=|\xi _6|$. We can confirm that the CCs of the five corresponding chordless cycles in Fig. 3(c) are precisely ones that are assigned above. For example, in $C_4$, there are four distinct fringe-trees whose molecular formulas are N, $\hbox {CH}_2$, CO, $\hbox {CH}_3$N, where we denote them by $\psi _1,\psi _2,\psi _3,\psi _4$, respectively. We have $\rm{mass}^*(\psi _1)=14$, $\rm{mass}^*(\psi _2)=12+2=14$, $\rm{mass}^*(\psi _3)=12+16=28$ and $\rm{mass}^*(\psi _4)=12+3+14=29$, where $\rm{mass}^*(\psi _1)=\rm{mass}^*(\psi _2)=14$, and hence $\xi (C_4)=(1,1,1,1,2,3)=\xi _4$ holds.

As we observed in Sect. ''Cycle-Configurations'', CC descriptors can distinguish how exteriors are attached to a chordless cycle in a chemical graph (e.g., meta/para-isomers of an aromatic ring), which is impossible by the original descriptors in the 2L model. Ring nodes are, however, not necessarily universal; there is a set of chordless cycles that cannot be represented by expanding ring nodes. For example:

(i) A pair of two chordless cycles that share exactly one point *v*.
(ii) A set of more than two chordless cycles that share one vertex or edge in common.

To infer ${{\mathbb {C}}}^\dagger$ that contains at least one of the above structures, one needs to make use of non-ring nodes/edges appropriately in the design of a seed tree. We note that, however, such chemical compounds are rather minor in conventional databases. See Table 1 again. For example, in PubChem, among 83,520,760 molecules that are acyclic or contain only chordless cycles whose lengths are within [4, 6], 80,842,345 molecules (96%) contain neither (i) nor (ii).

Table 2 shows a description of the specification $\sigma$ in the 2L$+$CC model. Besides the seed tree, the specification includes availability of chemical elements/configurations, lower/upper bounds on their numbers. One of the key strengths of the 2L$+$CC model is its ability to incorporate domain-specific chemical knowledge into the molecular inference process. First, the CC descriptors are available to capture important ring structural patterns, such as meta/para/ortho patterns on aromatic rings. By specifying the available configurations and imposing lower/upper bounds on their numbers as part of the specification $\sigma$, the framework allows the users to permit, restrict, or control the presence of specific structural motifs. Second, the modeling flexibility of the 2L$+$CC framework permits domain knowledge to be integrated through the design of the seed tree and the associated constraints in MILP formulations. For instance, users can define a benzene ring by including a ring node with all available CCs of length six in the seed tree and constraining which edges in the cycle are assigned as double bonds or single bonds. They can be easily implemented through additional linear constraints, and we regard this as a promising extension for future work. These features make the framework adaptable to chemically intuitive and commonly encountered molecular substructures.

**Table 2:** A description of specification $\sigma$ in the 2L$+$CC model (AC: adjacency-configuration; CC: cycle-configuration; EC: edge-configuration; FC: fringe-configuration)

Symbol	Definition
${{\mathcal {T}}}=(T;V^{\circ },E^{\circ })$	A seed tree
(A set of available chemical elements/configurations in $G_{{{\mathcal {T}}}}$)	
$\Lambda$	Chemical elements
$\Xi ^u$	CCs for $u\in V^{\circ }$, where $\xi \in \Xi ^u$ satisfies $c_{\min }\le |\xi |\le c_{\max }$
${{\mathcal {F}}}^u$	FCs for $u\in V(T)$
${\Gamma _\text{ac}^{\text{int}}}$	ACs on interior-edges
${\Gamma _\text{ac}^{\text{lf}}}$	ACs on leaf-edges
${\Gamma _\text{ec}^{\text{int}}}$	ECs on interior-edges
(Lower/upper bounds on the numbers in $G_{{{\mathcal {T}}}}$)	
${n_{\text{LB}}}, {n_{\text{UB}}}$	The number of non-hydrogen atoms
${\text{na}^{\text{int}}_{\text{LB}}}({\mathtt a}), {\text{na}^{\text{int}}_{\text{UB}}}({\mathtt a})$	The number of chemical elements ${\mathtt a}\in \Lambda$ in the interior
${\text{na}^{\text{ex}}_{\text{LB}}}({\mathtt a}), {\text{na}^{\text{ex}}_{\text{UB}}}({\mathtt a})$	The number of chemical elements ${\mathtt a}\in \Lambda$ in the exterior
${\text{na}_{\text{LB}}}({\mathtt a}), {\text{na}_{\text{UB}}}({\mathtt a})$	The number of chemical elements ${\mathtt a}\in \Lambda$ in $G_{{\mathcal {T}}}$
${\text{fc}_{\text{LB}}}(\psi ), {\text{fc}_{\text{UB}}}(\psi )$	The number of FCs $\psi \in {{\mathcal {F}}}^{{\mathcal {T}}}:=\bigcup _{u\in V(T)}{{\mathcal {F}}}^u$
${\text{ac}^{\text{int}}_{\text{LB}}}(\gamma ), {\text{ac}^{\text{int}}_{\text{UB}}}(\gamma )$	The number of ACs $\gamma \in {\Gamma _\text{ac}^{\text{int}}}$ in interior-edges
${\text{ac}^{\text{lf}}_{\text{LB}}}(\gamma ), {\text{ac}^{\text{lf}}_{\text{UB}}}(\gamma )$	The number of ACs $\gamma \in {\Gamma _\text{ac}^{\text{lf}}}$ in leaf-edges
${\text{ec}^{\text{int}}_{\text{LB}}}(\gamma ), {\text{ec}^{\text{int}}_{\text{UB}}}(\gamma )$	The number of ECs $\gamma \in {\Gamma _\text{ec}^{\text{int}}}$ in interior-edges

## Results and discussion

In this section, we describe experimental results on Stages 3 (ML) and 4 (MILP) in mol-infer. All experiments are conducted on a PC that carries Apple Silicon M1 CPU (3.2GHz) and 8GB main memory. All source codes are written in Python 3.12 with a machine learning library scikit-learn of version 1.5.0. The source codes and results are available at https://github.com/ku-dml/mol-infer/tree/master/2LCC.

### Experimental setup (stages 1 and 2)

We collected data sets for $27+17=44$ chemical properties that are shown in Table 3 and Table 4.

In 27 of the data sets, the property value $a({{\mathbb {C}}})$ of a chemical graph ${{\mathbb {C}}}$ is a real number, and hence the ML task of these 27 data sets in Stage 3 is regression. QM9 properties taken from [40] (i.e., Alpha, Cv, Gap, Homo, Lumo, mu and U0) share the same data set in common. This original data set contains more than $1.3\times 10^5$ molecules and we use a subset of $10^3$ molecules that are randomly selected.

In the other 17 data sets that derived from Tox21 collection [39], the property value $a({{\mathbb {C}}})$ of a chemical graph ${{\mathbb {C}}}$ is either negative or positive. Therefore, Stage 3 for these data sets is a binary classification task.

From the original data set, we exclude molecules that are not feasible in the 2L model (e.g., the chemical graph is not connected). Furthermore, we decide the set $\Lambda$ of available chemical elements for each property $\pi$, by which chemical graphs that contain rare chemical elements are eliminated.

Details of columns in Table 3 and Table 4 are described as follows.

- $\Lambda \setminus \{{\mathtt H}\}$: the set of available chemical elements except hydrogen, where
  $$\Lambda _1=\{{\mathtt C}_{(2)},{\mathtt C}_{(3)},{\mathtt C}_{(4)},{\mathtt C}_{(5)},{\mathtt O},{\mathtt N}_{(1)},{\mathtt N}_{(2)},{\mathtt N}_{(3)},{\mathtt F}\}$$; $$\Lambda _2=\{{\mathtt C},{\mathtt O},{\mathtt N},{\mathtt S}_{(2)},{\mathtt S}_{(6)},\texttt{Cl}\}$$; $$\Lambda _3=\{{\mathtt C},{\mathtt O}\}$$; $$\Lambda _4=\{{\mathtt C},{\mathtt O},{\mathtt N}\}$$; $$\Lambda _5=\{{\mathtt C}_{(2)},{\mathtt C}_{(3)},{\mathtt C}_{(4)},{\mathtt O},{\mathtt N}_{(2)},{\mathtt N}_{(3)},{\mathtt S}_{(2)},{\mathtt S}_{(4)}{\mathtt S}_{(6)},\texttt{Cl}\}$$; $$\Lambda _6=\{{\mathtt C},{\mathtt O},{\mathtt N},{\mathtt S}_{(2)},$$ $${\mathtt S}_{(4)},{\mathtt S}_{(6)},\texttt{Cl}\}$$; and $$\Lambda _7=\{{\mathtt C},{\mathtt O},\texttt{Si}\}$$. $$\Lambda _8=\{{\mathtt C},{\mathtt N}_{(2)},{\mathtt N}_{(3)},{\mathtt N}_{(4)},{\mathtt O}_{(1)},{\mathtt O}_{(2)},{\mathtt O}_{(3)}\}$$, $$\Lambda _9=\{{\mathtt C},{\mathtt N}_{(3)},{\mathtt N}_{(4)},{\mathtt O}_{(1)},{\mathtt O}_{(2)}\}$$.
- ${\underline{n}}$ and ${\overline{n}}$: the minimum and maximum values of the number of non-hydrogen atoms in ${{\mathbb {C}}}\in D_\pi$.
- $|D_\pi |$: the number of chemical graphs in the data set.
- $K_{\text {2L}}$ and $K_{\text {CC}}$: the number of 2L and CC descriptors extracted from $D_\pi$, respectively.

**Table 3:** Summary of regression data sets

$\pi$ (Description)	Refs.	$\Lambda \setminus \{{\mathtt H}\}$	${\underline{n}},{\overline{n}}$	$|D_\pi |$	$K_{\text {2L}}$	$K_{\text {CC}}$
Alpha (Isotropic polarizability)	[40]	$\Lambda _1$	6, 9	977	297	184
At (Autoignition temperature)	[41]	$\Lambda _2$	4, 85	448	255	65
Bhl (Biological half life)	[41]	$\Lambda _2$	5, 36	514	166	94
Bp (Boiling point)	[41]	$\Lambda _2$	4, 67	444	230	70
Cv (Heat Capacity at 298.15K)	[40]	$\Lambda _1$	6, 9	977	297	184
Dc (Dissociation constants)	[41]	$\Lambda _2$	5, 44	161	130	63
EDPA (Electron density on the most positive atom)	[42]	$\Lambda _3$	11, 16	52	64	6
Fp (Flash point in closed cup)	[41]	$\Lambda _2$	4, 67	424	229	70
Gap (Gap between Homo and Lumo)	[40]	$\Lambda _1$	6, 9	977	297	184
Hc (Heat of combustion)	[41]	$\Lambda _2$	4, 63	282	177	49
Homo (Energy of highest occupied molecular orbital)	[40]	$\Lambda _1$	6, 9	977	297	184
Hv (Heat of vaporization)	[41]	$\Lambda _4$	4, 16	95	105	16
IhcLiq (Isobaric heat capacities; liquid)	[43]	$\Lambda _4$	4, 78	770	256	74
IhcSol (Isobaric heat capacities; solid)	[43]	$\Lambda _5$	5, 70	668	228	118
KovRI (Kovats retention index)	[42]	$\Lambda _3$	11, 16	52	64	6
Kow (Octanol/water partition coefficient)	[41]	$\Lambda _4$	4, 58	684	223	117
Lp (Lipophilicity)	[40]	$\Lambda _6$	6, 74	936	231	178
Lumo (Energy of lowest occupied molecular orbital)	[40]	$\Lambda _1$	6, 9	977	297	184
Mp (Melting point)	[41]	$\Lambda _6$	4, 122	577	255	108
mu (Electric dipole moment)	[40]	$\Lambda _1$	6, 9	977	297	184
OptR (Optical rotation)	[41]	$\Lambda _4$	5, 44	147	107	55
Sl (Solubility)	[40]	$\Lambda _6$	4, 55	915	300	175
SurfT (Surface tension)	[44]	$\Lambda _7$	5, 33	247	128	22
U0 (Internal energy at 0K)	[40]	$\Lambda _1$	6, 9	977	297	184
Vd (Vapor density)	[41]	$\Lambda _4$	4, 30	474	214	53
Visc (Viscosity)	[45]	$\Lambda _7$	5, 36	282	126	22
Vp (Vapor pressure)	[41]	$\Lambda _{2}$	4, 5	482	238	96

**Table 4:** Summary of classification data sets

$\pi$ (Description)	Ref.	$\Lambda \setminus \{{\mathtt H}\}$	${\underline{n}},{\overline{n}}$	$|D_\pi |$	$K_{\text {2L}}$	$K_{\text {CC}}$
AhR (Aryl hydrocarbon Receptor)	[39]	$\Lambda _8$	5, 122	3875	506	333
AR (Androgen Receptor)	[39]	$\Lambda _8$	5, 122	4251	512	337
AR_LBD (Androgen Receptor Ligand-Binding Domain)	[39]	$\Lambda _8$	5, 122	3995	506	323
ARE (Androgen Response Element)	[39]	$\Lambda _8$	5, 122	3511	484	303
Aromatase (CYP19A)	[39]	$\Lambda _8$	5, 91	3469	487	320
ATAD5 (ATPase Family AAA Domain Containing 5)	[39]	$\Lambda _8$	5, 122	4161	514	331
ER (Estrogen Receptor)	[39]	$\Lambda _8$	5, 122	3712	489	323
ER_LBD (Estrogen Receptor Ligand-Binding Domain)	[39]	$\Lambda _8$	5, 122	4102	511	329
HSE (Heat Shock Factor Response Sequence)	[39]	$\Lambda _8$	5, 90	3808	497	312
MMP (Mitochondrial Membrane Potential)	[39]	$\Lambda _8$	5, 122	3527	478	320
MUTAG (MUTAGenetic effect)	[46]	$\Lambda _9$	11, 28	165	76	47
p53 (p53-mediated stress response pathway)	[39]	$\Lambda _8$	5, 122	4012	839	329
PPAR_$\gamma$ (Peroxisome proliferator-activated receptor $\gamma$)	[39]	$\Lambda _8$	5, 91	3835	828	325
PTC_FM (Predictive Toxicology Challenge Female Mice)	[47]	$\Lambda _4$	4, 50	141	195	69
PTC_FR (Predictive Toxicology Challenge Female Rats)	[47]	$\Lambda _4$	4, 50	146	130	73
PTC_MM (Predictive Toxicology Challenge Male Mice)	[47]	$\Lambda _4$	4, 50	130	122	63
PTC_MR (Predictive Toxicology Challenge Male Rats)	[47]	$\Lambda _4$	4, 50	144	127	67

**Table 5:** Summary of ML results on regression data sets. For each property π, an underlined value indicates the maximum over the 8 values (= 2 feature functions by 4 learning methods), bold-face (resp., *) indicates an R^2^ value that is larger at least by 0.02 (resp., 0.05) than the R^2^ value achieved by the other feature function and the same learning model

$\pi$ (Description)	2L model $f_{\text {2L}}$				2L$+$CC model $f_{\text {2L+CC}}$			
	LLR	DT	RF	ANN	LLR	DT	RF	ANN
Alpha (Isotropic polarizability)	0.961	0.769	0.856	0.934	0.961	0.784	0.875	0.923
At (Autoignition temperature)	0.388	0.368	0.380	0.474	0.405	0.379	**0.401**	**0.498**
Bhl (Biological half life)	0.483	0.401	0.555	0.629	**0.515**	*0.505	0.555	**0.677**
Bp (Boiling point)	0.663	0.729	0.805	0.720	0.**701**	0.728	0.824	*0.800
Cv (Heat Capacity at 298.15K)	0.970	0.805	0.911	0.943	0.979	**0.854**	0.911	0.946
Dc (Dissociation constants)	0.574	0.408	0.624	0.621	**0.607**	*0.476	0.629	*0.760
EDPA (Electron density on the most positive atom)	0.999	0.999	0.999	0.993	0.999	0.999	0.999	0.986
Fp (Flash point in closed cup)	0.570	0.572	0.748	0.744	0.564	*0.645	0.752	0.755
Gap (Gap between Homo and Lumo)	0.783	0.668	0.733	0.795	0.776	**0.712**	*0.786	0.801
Hc (Heat of combustion)	**0.951**	0.826	0.894	0.953	0.924	**0.857**	0.894	0.928
Homo (Energy of highest occupied molecular orbital)	0.707	0.391	0.556	0.689	0.703	**0.434**	*0.630	**0.722**
Hv (Heat of vaporization)	$-13.7$	0.128	$-0.058$	$-8.44$	*0.817	*0.554	*−**0.10**	*−3.89
IhcLiq (Isobaric heat capacities; liquid)	0.986	0.941	0.961	0.986	0.986	0.948	0.963	0.983
IhcSol (Isobaric heat capacities; solid)	0.981	0.903	0.952	0.973	0.983	0.908	0.954	0.966
KovRI (Kovats retention index)	0.676	0.352	0.688	0.727	*0.735	*0.644	0.688	*0.829
Kow (Octanol/water partition coefficient)	0.952	0.854	0.911	0.967	0.960	0.871	0.923	0.966
Lp (Lipophilicity)	0.840	0.598	0.756	0.858	0.855	0.616	**0.796**	0.864
Lumo (Energy of lowest occupied molecular orbital)	0.841	0.734	0.796	0.859	0.836	**0.759**	**0.842**	0.869
Mp (Melting point)	0.785	0.687	0.805	0.819	*0.836	**0.709**	**0.839**	*0.880
mu (Electric dipole moment)	0.365	0.351	0.433	0.384	0.368	**0.400**	**0.457**	0.395
OptR (Optical rotation)	0.822	0.846	**0.891**	0.918	*0.933	0.861	0.871	**0.957**
Sl (Solubility)	0.808	0.783	0.858	0.860	0.817	0.791	0.873	**0.880**
SurfT (Surface tension)	0.803	0.645	0.840	0.859	0.809	*0.714	0.840	0.869
U0 (Internal energy at 0K)	0.999	0.847	0.932	0.990	0.999	*0.910	0.932	0.982
Vd (Vapor density)	0.927	0.924	0.933	0.912	0.927	0.934	0.934	0.923
Visc (Viscosity)	0.893	0.860	0.909	0.929	0.894	0.866	0.910	0.937
Vp (Vapor pressure)	−0.1	0.771	0.857	$-100$	*0.115	*0.845	0.861	$-100$

**Table 6:** Summary of ML results on classification data sets. For each property π, an underlined value indicates the maximum over the 8 values (= 2 feature functions by 4 learning methods), bold-face (resp., *) indicates a BACC value that is larger at least by 0.02 (resp., 0.05) than the BACC value achieved by the other feature function and the same learning model

$\pi$ (Description)	2L model $f_{\text {2L}}$				2L$+$CC model $f_{\text {2L+CC}}$			
	LLR	DT	RF	ANN	LLR	DT	RF	ANN
AhR (Aryl hydrocarbon Receptor)	0.670	0.729	0.727	0.820	*0.820	0.732	0.726	0.833
AR (Androgen Receptor)	0.740	0.708	0.724	0.750	0.756	**0.728**	0.732	0.766
AR_LBD (Androgen Receptor Ligand-Binding Domain)	0.810	0.818	0.805	0.710	*0.872	0.814	**0.825**	*0.895
ARE (Androgen Response Element)	0.730	0.676	0.664	0.740	0.725	0.678	0.670	0.747
Aromatase (CYP19A)	0.730	0.612	0.571	0.730	0.743	0.610	0.573	0.749
ATAD5 (ATPase Family AAA Domain Containing 5)	0.740	0.645	0.626	0.760	**0.769**	**0.673**	0.630	**0.795**
ER (Estrogen Receptor)	0.700	0.647	0.661	0.710	0.710	0.660	0.670	0.722
ER_LBD (Estrogen Receptor Ligand-Binding Domain)	0.770	0.727	0.728	0.780	**0.800**	0.732	0.734	**0.800**
HSE (Heat Shock Factor Response Sequence)	0.660	0.584	0.554	0.630	0.658	0.584	0.557	**0.690**
MMP (Mitochondrial Membrane Potential)	0.800	0.747	0.711	0.830	**0.820**	0.749	**0.747**	0.844
MUTAG (MUTAGenetic effect)	0.880	0.790	0.907	0.920	**0.909**	*0.900	0.909	0.932
p53 (p53-mediated stress response pathway)	0.760	0.652	0.608	0.760	0.760	0.647	0.608	0.772
PPAR_$\gamma$ (Peroxisome proliferator-activated receptor $\gamma$)	0.720	0.624	0.598	0.720	0.731	0.626	0.598	*0.773
PTC_FM (Predictive Toxicology Challenge Female Mice)	0.540	0.674	0.711	0.600	*0.612	0.670	0.708	*0.697
PTC_FR (Predictive Toxicology Challenge Female Rats)	0.600	0.613	0.623	0.630	0.594	0.611	0.621	*0.671
PTC_MM (Predictive Toxicology Challenge Male Mice)	0.550	0.570	0.657	0.660	*0.604	*0.638	0.663	0.641
PTC_MR (Predictive Toxicology Challenge Male Rats)	0.550	0.590	0.642	0.620	**0.578**	**0.619**	0.653	*0.712

### ML experiments (stage 3)

For each property $\pi$, we convert the data set $D_\pi$ into the set $f(D_\pi )$ of numerical vectors by using a feature function $f:{{\mathcal {G}}}\rightarrow {{\mathbb {R}}}^K$. For *f*, we use $f=f_{\text {2L}}$ and $f=f_{\text {2L+CC}}$, where $f_{\text {2L+CC}}$ is a feature function such that $f_{\text {2L+CC}}({{\mathbb {C}}})=(f_{\text {2L}}({{\mathbb {C}}}),f_{\text {CC}}({{\mathbb {C}}}))$. The purpose of the comparison is to show that CC descriptors can extract useful information for ML. The number $K_{\text {CC}}$ of CC descriptors is at most 70% of the number $K_{\text {2L}}$ of 2L descriptors for all data sets, as shown in Table 3 and Table 4.

For $\pi$, let $D\subseteq D_\pi$ be a subset of the data set. To evaluate a prediction function $\eta :{{\mathbb {R}}}^K\rightarrow {{\mathbb {R}}}$ on *D*, we employ the determination of coefficient ($\hbox {R}^2$) for regression task, which is defined to be

$$\begin{aligned} \text {R}^2(\eta ,D)\triangleq 1-\frac{\sum _{{{\mathbb {C}}}\in D}(a({{\mathbb {C}}})-\eta (f({{\mathbb {C}}})))^2}{\sum _{{{\mathbb {C}}}\in D}(a({{\mathbb {C}}})-{\tilde{a}})^2}\text { for }{\tilde{a}}=\frac{1}{|D|}\sum _{{{\mathbb {C}}}\in D}a({{\mathbb {C}}}). \end{aligned}$$

For a binary classification case, let us denote $D_i:=\{{{\mathbb {C}}}\in D\mid a({{\mathbb {C}}})=i\}$ for $i=0,1$, TP($\eta ,D$)$:=|\{{{\mathbb {C}}}\in D_1\mid \eta (f({{\mathbb {C}}}))=1\}|$, FN($\eta ,D$)$:=|\{{{\mathbb {C}}}\in D_1\mid \eta (f({{\mathbb {C}}}))=0\}|$, TN($\eta ,D$)$:=|\{{{\mathbb {C}}}\in D_0\mid \eta (f({{\mathbb {C}}}))=0\}|$, and FP($\eta ,D$)$:=|\{{{\mathbb {C}}}\in D_0\mid \eta (f({{\mathbb {C}}}))=1\}|$. To evaluate a prediction function $\eta :{{\mathbb {R}}}^K\rightarrow \{0,1\}$ on *D*, we employ the balanced accuracy (BACC), which is defined to be

$$\begin{aligned} \text {BACC}(\eta ,D)\triangleq \frac{1}{2}\big (\frac{\text {TP}(\eta ,D)}{\text {TP}(\eta ,D)+\text {FN}(\eta ,D)} + \frac{\text {TN}(\eta ,D)}{\text {TN}(\eta ,D)+\text {FP}(\eta ,D)} \big ). \end{aligned}$$

We construct prediction functions based on Lasso linear regression (LLR) [48], decision tree (DT) [49], artificial neural networks (ANN) [50] and random forest (RF) [51]. We evaluate the performance of each learning model by means of 10 repetitions of 5-fold cross validation. Specifically, for each property $\pi$, we divide the data set $D_\pi$ into 5 subsets randomly, say $D_{\pi ,1},\dots ,D_{\pi ,5}$, so that $|D_{\pi ,i}|-|D_{\pi ,j}|\le 1$ holds for $i,j=1,2,\dots ,5$. For each $i=1,2,\dots ,5$, we construct a prediction function $\eta$ from a subset $D_\pi \setminus D_{\pi ,i}$ as the training set, based on different data sets, evaluate $\text {R}^2(\eta ,D_{\pi ,i})$ or $\text {BACC}(\eta ,D_{\pi ,i})$ on the remaining subset $D_{\pi ,i}$ as the test set. We take as the evaluation criterion the median of $5\times 10=50$ values of $\hbox {R}^2$ and BACC observed over 10 repetitions of 5-fold cross validation. It should be emphasized that for binary classification tasks, the prediction function constructed by DT, RF and ANN can directly classify the property class as negative or positive, but when the prediction function is constructed by LLR, the predicted value obtained by the test data is a real number, which cannot be used directly to classify the property class. Let us denote the training set by $D_t$ and denote $C_1$ and $C_0$ as the number of positive and negative molecules in the training set, respectively. We use $\theta$ to denote the classification threshold, which is defined to be

$$\begin{aligned} \theta \triangleq \underset{r \in R}{\arg \max }\ \, \widetilde{\text {BACC}}(\eta ,r), \end{aligned}$$

where $$R\triangleq \{\eta ({{\mathbb {C}}}) \mid {{\mathbb {C}}}\in D_t\}$$ and

$$\begin{aligned} \widetilde{\text {BACC}}(\eta ,r)\triangleq \frac{1}{2}\big (\frac{\widetilde{\text {TP}}(\eta ,r)}{C_1} + \frac{\widetilde{\text {TN}}(\eta ,r)}{C_0} \big ), \end{aligned}$$

where $\widetilde{\text {TP}}(\eta ,r) \triangleq |\{{{\mathbb {C}}}\in D_t \mid a({{\mathbb {C}}})=1,\eta ({{\mathbb {C}}}) \ge r \}|$ and $\widetilde{\text {TN}}(\eta ,r) \triangleq |\{{{\mathbb {C}}}\in D_t \mid a({{\mathbb {C}}})=0,\eta ({{\mathbb {C}}}) \le r \}|$. When it holds that $\theta \le \eta ({{\mathbb {C}}})$, we regard the property class of ${{\mathbb {C}}}$ as positive.

We show the results in Table 5 and Table 6. We may say that we can construct a good prediction function for many data sets; for the regression data sets, in 19 (resp., 11) out of the 27 data sets, $\hbox {R}^2$ over 0.8 (resp., 0.9) is achieved. We observe that, for some data sets, there is a learning model that is not suitable. For example, LLR and ANN perform poorly for Vp, regardless of feature functions. Notably, ANN performs extremely poorly, yielding an $\hbox {R}^2$ value of -100, indicating its ineffectiveness for this data set. In contrast, DT and RF are relatively good. For the classification data sets, we can see that the performance is rather good, in 13 (resp., 5) out of the 17 data sets since BACC score over 0.7 (resp., 0.8) is achieved.

Let us compare two feature functions $f_{\text {2L}}$ and $f_{\text {2L+CC}}$ for regression task and classification task respectively. For each property $\pi$, an underlined value indicates the maximum over the 8 values ($=$ 2 feature functions by 4 learning models). For regression task, the maximum is achieved for only 5 properties when $f=f_{\text {2L}}$, whereas it is up to 26 properties when $f=f_{\text {2L+CC}}$. For classification task, the maximum is achieved for 1 properties when $f=f_{\text {2L}}$, whereas it is up to 16 properties when $f=f_{\text {2L+CC}}$. In both Table 5 and Table 6, bold-face (resp., *) indicates an $\hbox {R}^2$ value or a BACC value that is larger at least by 0.02 (resp., 0.05) than the $\hbox {R}^2$ value or a BACC value achieved by the other feature function and the same learning model. For example, for At, the $\hbox {R}^2$ value 0.401 for $f_{\text {2L+CC}}$ and RF is bold since it is larger than 0.379 for $f_{\text {2L}}$ and RF by $0.401-0.379=0.022>0.02$. In Table 5, a bold value (resp., *) appears only twice (resp., nowhere) when $f=f_{\text {2L}}$, whereas it appears 41 (resp., 21) times when $f=f_{\text {2L+CC}}$. In Table 6, a bold value (resp., *) appears nowhere (resp., nowhere) when $f=f_{\text {2L}}$, whereas it appears 24 (resp., 10) times when $f=f_{\text {2L+CC}}$.

We conclude that, in the 2L model, the learning performance of a prediction function can be improved by introducing CC descriptors.

### Inference experiments (stage 4)

For a given property, after deciding two reals ${\underline{y}}^*,{\overline{y}}^*\in {{\mathbb {R}}}$ and a specification $\sigma$, we solve the MILP for inferring a chemical graph ${{\mathbb {C}}}^\dagger$. Recall that the MILP consists of two families of constraints, that is $({{\mathcal {C}}}_1)$ ${\underline{y}}^*\le \eta (x)\le {\overline{y}}^*$ and $x=f({{\mathbb {C}}}^\dagger )$; and $({{\mathcal {C}}}_2)$ ${{\mathbb {C}}}\in {{\mathcal {G}}}_\sigma$. In this experiment, for the regression datasets, we employ a hyperplane that is learned by LLR for the prediction function $\eta$. A hyperplane is a prediction function that is represented by a pair $(w,b)\in {{\mathbb {R}}}^K\times {{\mathbb {R}}}$ and predicts the property value of a feature vector $x\in {{\mathbb {R}}}^K$ by $w(1)x(1)+\dots +w(K)x(K)+b$. Hence, the constraint ${\underline{y}}^*\le \eta (x)\le {\overline{y}}^*$ in ${{\mathcal {C}}}_1$ is represented by

$$\begin{aligned} {\underline{y}}^*\le \sum _{j=1}^Kw(j)x(j)+b\le {\overline{y}}^*, \end{aligned}$$

where use of a hyperplane in mol-infer was proposed in [29]. In classification tasks, we also use the hyperplane constructed by LLR as the prediction function. When we aim to infer molecules that exhibit positive for the target property, the constraint in ${{\mathcal {C}}}_1$ is represented by

$$\begin{aligned} \theta \le \sum _{j=1}^Kw(j)x(j)+b. \end{aligned}$$

When we aim to infer molecules that exhibit negative for the target property, the constraint in ${{\mathcal {C}}}_1$ is represented by

$$\begin{aligned} \sum _{j=1}^Kw(j)x(j)+b \le \theta , \end{aligned}$$

where $\theta$ is the classification threshold. For the other constraints, see Appendix B.

**Fig. 4:**
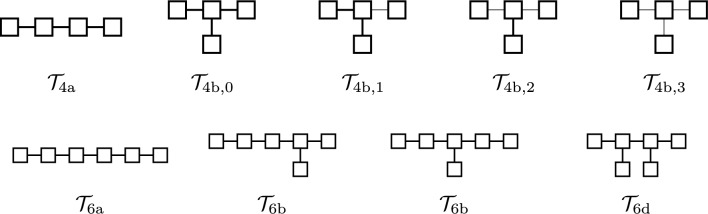
Seed trees for the inference experiments: All nodes are ring nodes. A ring edge (resp., a non-ring edge) is depicted by a thick (resp., thin) line

In regression data sets, we take up two properties Kow and OptR, for which LLR achieves $\hbox {R}^2$ over 0.9. In classification data sets, we take up two properties AhR and AR_LBD, for which LLR achieves BACC score over 0.8. We consider 10 specifications that have seed trees non-isomorphic to each other, where 9 out of the 10 seed trees are shown in Fig. 4. These seed trees are introduced to observe how computation time changes with respect to the number of nodes; the number of ring edges; and the tree structure. The last seed tree is the one in Fig. 3(a). We denote this seed tree by ${{\mathcal {T}}}_{5^*}$. In each of the 10 specifications, we set other parameters (see Table 2) than the seed tree sufficiently large to the extent of the data set $D_\pi$. For example, we set ${{\mathcal {F}}}^u:={{\mathcal {F}}}(D_\pi )$ for every $u\in T$, that is, all fringe trees that appear in $D_\pi$ are available to *u*.

We solve the MILP by utilizing CPLEX [52] version 22.1.1.0. We summarize statistics in Tables 7, 8, 9,10. The meanings of columns in the tables are described as follows.

- #V and #C: the number of variables and constraints in MILP, respectively.
- IP time: the computation time (in seconds) taken to solve the MILP.
- $n(G^\dagger )$: the number of non-hydrogen atoms in the inferred chemical graph $G^\dagger$.
- $\eta (f(G^\dagger ))$: an estimated property value of $G^\dagger$ given by the prediction function $\eta$ and the feature vector $f(G^\dagger )$, where $f=f_{\text {2L+CC}}$.

**Table 7:** Statistics of MILPs for Kow: ${\underline{y}}^*$ and ${\overline{y}}^*$ are set to −7.53 and 15.60, respectively

Seed tree		#V	#C	IP time	$n(G^\dagger )$	$\eta (f(G^\dagger ))$
${{\mathcal {T}}}_{\text {4a}}$		15139	18145	5.2	18	3.30
${{\mathcal {T}}}_{\text {4b,0}}$		15139	18145	4.6	18	3.64
${{\mathcal {T}}}_{\text {4b,1}}$		14921	13843	4.1	18	3.64
${{\mathcal {T}}}_{\text {4b,2}}$		14703	9541	38.4	25	−5.38
${{\mathcal {T}}}_{\text {4b,3}}$		14485	5239	7.0	44	0.19
${{\mathcal {T}}}_{\text {6a}}$		21743	27843	7.8	26	4.42
${{\mathcal {T}}}_{\text {6b}}$		21743	27843	11.1	26	4.76
${{\mathcal {T}}}_{\text {6c}}$		21743	27843	9.4	26	4.76
${{\mathcal {T}}}_{\text {6d}}$		21743	27843	7.9	26	5.09
${{\mathcal {T}}}_{5^*}$		19923	10329	59.0	46	−2.81

**Table 8:** Statistics of MILPs for OptR: ${\underline{y}}^*$ and ${\overline{y}}^*$ are set to −117.0 and 165.0, respectively

Seed tree		#V	#C	IP time	$n(G^\dagger )$	$\eta (f(G^\dagger ))$
${{\mathcal {T}}}_{\text {4a}}$		8229	11853	12.1	21	−53.06
${{\mathcal {T}}}_{\text {4b,0}}$		8229	11853	13.0	25	−102.25
${{\mathcal {T}}}_{\text {4b,1}}$		8122	9327	8.2	28	−48.79
${{\mathcal {T}}}_{\text {4b,2}}$		8015	6801	28.8	30	45.47
${{\mathcal {T}}}_{\text {4b,3}}$		7908	4275	13.1	25	26.16
${{\mathcal {T}}}_{\text {6a}}$		11587	17875	13.7	34	−54.39
${{\mathcal {T}}}_{\text {6b}}$		11587	17875	27.7	34	134.51
${{\mathcal {T}}}_{\text {6c}}$		11587	17875	15.9	21	−92.59
${{\mathcal {T}}}_{\text {6d}}$		11587	17875	117.8	37	−110.17
${{\mathcal {T}}}_{5^*}$		10652	7527	48.7	36	−103.90

**Table 9:** Statistics of MILPs for AhR: Targets are set to Positive

Seed tree		#V	#C	IP time	$n(G^\dagger )$	$\eta (f(G^\dagger ))$
${{\mathcal {T}}}_{\text {4a}}$		15529	23095	48.6	18	Positive
${{\mathcal {T}}}_{\text {4b,0}}$		14583	6451	1.5	19	Positive
${{\mathcal {T}}}_{\text {4b,1}}$		36602	33490	118.3	19	Positive
${{\mathcal {T}}}_{\text {4b,2}}$		36602	33490	470.5	22	Positive
${{\mathcal {T}}}_{\text {4b,3}}$		36129	25168	89.1	23	Positive
${{\mathcal {T}}}_{\text {6a}}$		53130	51660	374.3	26	Positive
${{\mathcal {T}}}_{\text {6b}}$		53130	51660	428.0	28	Positive
${{\mathcal {T}}}_{\text {6c}}$		53130	51660	191.6	30	Positive
${{\mathcal {T}}}_{\text {6d}}$		53130	51660	1048.9	35	Positive
${{\mathcal {T}}}_{5^*}$		48337	17850	52.8	61	Positive

**Table 10:** Statistics of MILPs for AR_LBD: Targets are set to Positive

Seed tree		#V	#C	IP time	$n(G^\dagger )$	$\eta (f(G^\dagger ))$
${{\mathcal {T}}}_{\text {4a}}$		15476	23096	17.5	38	Positive
${{\mathcal {T}}}_{\text {4b,0}}$		14519	6402	3.6	36	Positive
${{\mathcal {T}}}_{\text {4b,1}}$		36072	33457	405.3	28	Positive
${{\mathcal {T}}}_{\text {4b,2}}$		36072	33457	14.6	36	Positive
${{\mathcal {T}}}_{\text {4b,3}}$		35598	25119	396.1	29	Positive
${{\mathcal {T}}}_{\text {6a}}$		44236	50993	3.4	Infeasible	
${{\mathcal {T}}}_{\text {6b}}$		44236	50993	3.5	Infeasible	
${{\mathcal {T}}}_{\text {6c}}$		44236	50993	3.4	Infeasible	
${{\mathcal {T}}}_{\text {6d}}$		44236	50993	26.4	52	Positive
${{\mathcal {T}}}_{5^*}$		47670	17775	56.0	58	Positive

We highlight the practical efficiency of our MILP formulation with CC descriptors, as shown in Tables 7 to 10. Across a range of seed trees and properties, chemical graphs with up to 50 non-hydrogen atoms were inferred within a practical time—most of the formulations were solved in less than one minute, a few were solved in about five minutes and only one was solved in around 18 min. There is a tendency such that computation time is longer when there are more #V/#C (i.e., the numbers of variables/constraints in MILP) with some exceptions. For example, for Kow, the case of ${{\mathcal {T}}}_{\text {4b,2}}$ takes 38.4 s, which is much more than the cases where there are six ring nodes. Concerning #V/#C, the more the ring nodes, the more they become. The #V/#C are equal between seed trees if they have the same numbers of ring nodes/edges; e.g., #V/#C are equal between ${{\mathcal {T}}}_{\text {4a}}$ and ${{\mathcal {T}}}_{\text {4b,0}}$. These results indicate that adding CC descriptors does not lead to too much computational overhead in practice.

**Fig. 5:**
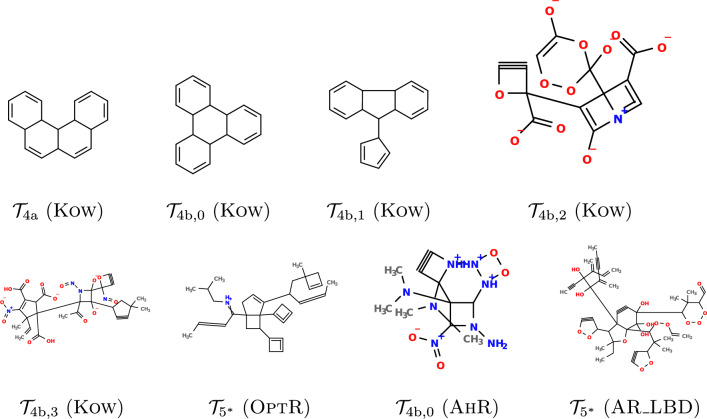
Inferred chemical graphs

We also show some of the inferred chemical graphs in Fig. 5. As expected, ring nodes in the seed trees are expanded to cycles in the chemical graphs. We acknowledge that some of the inferred graphs seem unstable and hard to synthesize (e.g., 4-cycles or ionized elements). This is a common issue in molecular inference studies [53]. We can prevent MILP from using such structures by setting specifications appropriately. Addressing this issue is left as future work.

## Conclusions

In this paper, we proposed a new family of descriptors, cycle-configurations, that can be used in the standard 2L model of mol-infer. We introduced the definition in Sect. '“Cycle-Configurations'' and described how we deal with them in the MILP in Sect. “MILP Formulation for 2L+CC Model''. Then in Sect. “Results and Discussion'', we demonstrated that the performance of a prediction function is improved in many cases when we introduce CC descriptors. We also showed that a chemical graph with up to 50 non-hydrogen atoms can be inferred in a practical time.

The 2L+CC model can be extended further in the similar way as the 2L model. Specifically, we can enumerate isomers of the inferred graph by dynamic programming [54] or generate “close” compounds in the sense of property values by a grid neighborhood approach [55]. Note that the constraint ${{\mathcal {C}}}_1$ of the MILP can contain multiple prediction functions for multiple properties, as is done in [29], where we have included a single property in this paper for simplicity. Besides, we may apply the 2L+CC model to inference of polymers [36]. These are left for future work.

## Data Availability

All datasets, source codes and results are available at https://github.com/ku-dml/mol-infer/tree/master/2LCC.

## Appendix A a full description of descriptors in the 2L model

Associated with the two functions $\alpha$ and $\beta$ in a chemical graph ${{\mathbb {C}}}=(H,\alpha ,\beta )$, we introduce functions $\text{ac}: V(E)\rightarrow (\Lambda {\setminus }\{{\mathtt H}\})\times (\Lambda {\setminus }\{{\mathtt H}\})\times [1,3]$, $\text{cs}: V(E)\rightarrow (\Lambda {\setminus }\{{\mathtt H}\})\times [1,4]$ and $\text{ec}: V(E)\rightarrow ((\Lambda {\setminus }\{{\mathtt H}\})\times [1,4])\times ((\Lambda {\setminus }\{{\mathtt H}\})\times [1,4])\times [1,3]$ in the following.

To represent a feature of the exterior of ${{\mathbb {C}}}$, a chemical rooted tree in ${\mathcal {T}}({{\mathbb {C}}})$ is called a *fringe-configuration* of ${{\mathbb {C}}}$.

We also represent leaf-edges in the exterior of ${{\mathbb {C}}}$. For a leaf-edge $uv\in E(\langle {{\mathbb {C}}}\rangle )$ with $\deg _{\langle {{\mathbb {C}}}\rangle }(u)=1$, we define the *adjacency-configuration* of *e* to be an ordered tuple $(\alpha (u),\alpha (v),\beta (uv))$. Define

$$\begin{aligned} \Gamma _\text{ac}^\text{lf}\triangleq \{({\mathtt a},{\mathtt b},m)\mid {\mathtt a},{\mathtt b}\in \Lambda , m\in [1,\min \{\text{val}({\mathtt a}),\rm{val}({\mathtt b})\}]\} \end{aligned}$$

as a set of possible adjacency-configurations for leaf-edges.

To represent a feature of an interior-vertex $v\in V^{\rm int}({{\mathbb {C}}})$ such that $\alpha (v)={\mathtt a}$ and $\deg _{\langle {{\mathbb {C}}}\rangle }(v)=d$ (i.e., the number of non-hydrogen atoms adjacent to *v* is *d*) in a chemical graph ${{\mathbb {C}}}=(H,\alpha ,\beta )$, we use a pair $({\mathtt a}, d)\in (\Lambda {\setminus }\{\texttt {H}\})\times [1,4]$, which we call the *chemical symbol* ${\rm cs}(v)$ of the vertex *v*. We treat $({\mathtt a}, d)$ as a single symbol ${\mathtt a}d$, and define $\Lambda _{\rm{dg}}$ to be the set of all chemical symbols $\mu ={\mathtt a}d\in (\Lambda {\setminus }\{\texttt{H}\})\times [1,4]$.

We define a method for featuring interior-edges as follows. Let $e=uv\in E^{\rm int}({{\mathbb {C}}})$ be an interior-edge $e=uv\in E^{\rm int}({{\mathbb {C}}})$ such that $\alpha (u)={\mathtt a}$, $\alpha (v)={\mathtt b}$ and $\beta (e)=m$ in a chemical graph ${{\mathbb {C}}}=(H,\alpha ,\beta )$. To feature this edge *e*, we use a tuple $({\mathtt a},{\mathtt b},m)\in (\Lambda {\setminus }\{\texttt {H}\}) \times (\Lambda {\setminus }\{\texttt {H}\})\times [1,3]$, which we call the *adjacency-configuration* ${\rm ac}(e)$ of the edge *e*. We introduce a total order < over the elements in $\Lambda$ to distinguish between $({\mathtt a},{\mathtt b}, m)$ and $({\mathtt b},{\mathtt a}, m)$ $({\mathtt a}\ne {\mathtt b})$ notationally. For a tuple $\nu =({\mathtt a},{\mathtt b}, m)$, let ${\overline{\nu }}$ denote the tuple $({\mathtt b},{\mathtt a}, m)$.

Let $e=uv\in E^{\rm int}({{\mathbb {C}}})$ be an interior-edge $e=uv\in E^{\rm int}({{\mathbb {C}}})$ such that ${\rm cs}(u)=\mu$, ${\rm cs}(v)=\mu '$ and $\beta (e)=m$ in a chemical graph ${{\mathbb {C}}}=(H,\alpha ,\beta )$. To feature this edge *e*, we use a tuple $(\mu ,\mu ',m)\in \Lambda _{\rm{dg}}\times \Lambda _{\rm{dg}}\times [1,3]$, which we call the *edge-configuration* ${\rm ec}(e)$ of the edge *e*.

We introduce a total order < over the elements in $\Lambda _{\rm{dg}}$ to distinguish between $(\mu ,\mu ', m)$ and $(\mu ', \mu , m)$ $(\mu \ne \mu ')$ notationally. For a tuple $\gamma =(\mu ,\mu ', m)$, let ${\overline{\gamma }}$ denote the tuple $(\mu ', \mu , m)$.

Let $\pi$ be a chemical property for which we will construct a prediction function $\eta$ from a feature vector $f({{\mathbb {C}}})$ of a chemical graph ${{\mathbb {C}}}$ to a predicted value $y\in {\mathbb {R}}$ for the chemical property of ${{\mathbb {C}}}$.

We first choose a set $\Lambda$ of chemical elements and then collect a data set $D_{\pi }$ of chemical compounds *C* whose chemical elements belong to $\Lambda$, where we regard $D_{\pi }$ as a set of chemical graphs ${{\mathbb {C}}}$ that represent the chemical compounds *C* in $D_{\pi }$. To define the interior/exterior of chemical graphs ${{\mathbb {C}}}\in D_{\pi }$, we next choose a branch-parameter ${\rho }$, where we recommend ${\rho }=2$.

Let $\Lambda ^{\rm int}(D_\pi )\subseteq \Lambda$ (resp., $\Lambda ^{\rm ex}(D_\pi )\subseteq \Lambda$) denote the set of chemical elements used in the set $V^{\rm int}({{\mathbb {C}}})$ of interior-vertices (resp., the set $V^{\rm ex}({{\mathbb {C}}})$ of exterior-vertices) of ${{\mathbb {C}}}$ over all chemical graphs ${{\mathbb {C}}}\in D_\pi$, and $\Gamma ^{\rm int}(D_\pi )$ denote the set of edge-configurations used in the set $E^{\rm int}({{\mathbb {C}}})$ of interior-edges in ${{\mathbb {C}}}$ over all chemical graphs ${{\mathbb {C}}}\in D_\pi$. Let ${\mathcal {F}}(D_\pi )$ denote the set of chemical rooted trees $\psi$ r-isomorphic to a chemical rooted tree in ${\mathcal {T}}({{\mathbb {C}}})$ over all chemical graphs ${{\mathbb {C}}}\in D_\pi$, where possibly a chemical rooted tree $\psi \in {\mathcal {F}}(D_\pi )$ consists of a single chemical element ${\mathtt a}\in \Lambda {\setminus } \{\texttt {H}\}$.

We define an integer encoding of a finite set *A* of elements to be a bijection $\pi : A \rightarrow [1, |A|]$, where we denote by [*A*] the set [1, |*A*|] of integers. Introduce an integer coding of each of the sets $\Lambda ^{\rm int}(D_\pi )$, $\Lambda ^{\rm ex}(D_\pi )$, $\Gamma ^{\rm int}(D_\pi )$ and ${\mathcal {F}}(D_\pi )$. Let $[{\mathtt a}]^{\rm int}$ (resp., $[{\mathtt a}]^{\rm ex}$) denote the coded integer of an element ${\mathtt a}\in \Lambda ^{\rm int}(D_\pi )$ (resp., ${\mathtt a}\in \Lambda ^{\rm ex}(D_\pi )$), $[\gamma ]$ denote the coded integer of an element $\gamma$ in $\Gamma ^{\rm int}(D_\pi )$ and $[\psi ]$ denote an element $\psi$ in ${\mathcal {F}}(D_\pi )$.

Over 99% of chemical compounds ${{\mathbb {C}}}$ with up to 100 non-hydrogen atoms in PubChem have degree at most 4 in the hydrogen-suppressed graph $\langle {{\mathbb {C}}}\rangle$ [56]. We assume that a chemical graph ${{\mathbb {C}}}$ treated in this paper satisfies $\deg _{\langle {{\mathbb {C}}}\rangle }(v)\le 4$ in the hydrogen-suppressed graph $\langle {{\mathbb {C}}}\rangle$.

In our model, we use an integer $\rm{mass}^*({\mathtt a})=\lfloor 10\cdot \rm{mass}({\mathtt a})\rfloor$, for each ${\mathtt a}\in \Lambda$.

For a chemical property $\pi$, we define a set $D_\pi ^{(1)}$ of descriptors of a chemical graph ${{\mathbb {C}}}=(H,\alpha ,\beta )\in D_{\pi }$ to be the following non-negative values ${\rm dcp}_i({{\mathbb {C}}})$, $i\in [1,K_{\text {2L}}]$, where $K_{\text {2L}}= 14+ |\Lambda ^{\rm int}(D_\pi )|+|\Lambda ^{\rm ex}(D_\pi )| +|\Gamma ^{\rm int}(D_\pi )|+|{\mathcal {F}}(D_\pi )|+|\Gamma _{\rm ac}^{\rm lf}|$.

1. $\rm{dcp}_1({{\mathbb {C}}})$: the number $|V(H)|-|V_{{\mathtt H}}|$ of non-hydrogen atoms in ${{\mathbb {C}}}$.
2. $\rm{dcp}_2({{\mathbb {C}}})$: the rank of ${{\mathbb {C}}}$ (i.e., the minimum number of edges to be removed to make the graph acyclic).
3. $\rm{dcp}_3({{\mathbb {C}}})$: the number $|V^{\rm int}({{\mathbb {C}}})|$ of interior-vertices in ${{\mathbb {C}}}$.
4. $\rm{dcp}_4({{\mathbb {C}}})$: the average $\overline{\rm{ms}}({{\mathbb {C}}})$ of $\hbox {mass}^*$ over all atoms in ${{\mathbb {C}}}$;
  i.e., $$\overline{\rm{ms}}({{\mathbb {C}}})\triangleq \frac{1}{|V(H)|}\sum _{v\in V(H)}{\rm mass}^*(\alpha (v))$$.
5. ${\rm dcp}_i({{\mathbb {C}}})$, $i=4+d, d\in [1,4]$: the number ${\rm dg}_d^{\overline{\texttt {H}}} ({{\mathbb {C}}})$ of non-hydrogen vertices $v\in V(H){\setminus } V_{{\mathtt H}}$ of degree $\deg _{\langle {{\mathbb {C}}}\rangle }(v)=d$ in the hydrogen-suppressed chemical graph $\langle {{\mathbb {C}}}\rangle$.
6. ${\rm dcp}_i({{\mathbb {C}}})$, $i=8+d, d\in [1,4]$: the number ${\rm dg}_d^{\rm int}({{\mathbb {C}}})$ of interior-vertices of interior-degree $\deg _{{{\mathbb {C}}}^{\rm int}}(v)=d$ in the interior ${{\mathbb {C}}}^{\rm int}=(V^{\rm int}({{\mathbb {C}}}),E^{\rm int}({{\mathbb {C}}}))$ of ${{\mathbb {C}}}$.
7. ${\rm dcp}_i({{\mathbb {C}}})$, $i=12+m$, $m\in [2,3]$: the number ${\rm bd}_m^{\rm int}({{\mathbb {C}}})$ of interior-edges with bond multiplicity *m* in ${{\mathbb {C}}}$; i.e., ${\rm bd}_m^{\rm int}({{\mathbb {C}}})\triangleq |\{e\in E^{\rm int}({{\mathbb {C}}})\mid \beta (e)=m\}|$.
8. ${\rm dcp}_i({{\mathbb {C}}})$, $i=14+[{\mathtt a}]^{\rm int}$, ${\mathtt a}\in \Lambda ^{\rm int}(D_\pi )$: the frequency ${\rm na}_{\mathtt a}^{\rm int}({{\mathbb {C}}})=|V_{\mathtt a}({{\mathbb {C}}})\cap V^{\rm int}({{\mathbb {C}}}) |$ of chemical element ${\mathtt a}$ in the set $V^{\rm int}({{\mathbb {C}}})$ of interior-vertices in ${{\mathbb {C}}}$.
9. ${\rm dcp}_i({{\mathbb {C}}})$, $i=14+|\Lambda ^{\rm int}(D_\pi )|+[{\mathtt a}]^{\rm ex}$, ${\mathtt a}\in \Lambda ^{\rm ex}(D_\pi )$: the frequency ${\rm na}_{\mathtt a}^{\rm ex}({{\mathbb {C}}})=|V_{\mathtt a}({{\mathbb {C}}})\cap V^{\rm ex}({{\mathbb {C}}}) |$ of chemical element ${\mathtt a}$ in the set $V^{\rm ex}({{\mathbb {C}}})$ of exterior-vertices in ${{\mathbb {C}}}$.
10. ${\rm dcp}_i({{\mathbb {C}}})$, $i=14+|\Lambda ^{\rm int}(D_\pi )|+|\Lambda ^{\rm ex}(D_\pi )|+ [\gamma ]$, $\gamma \in \Gamma ^{\rm int}(D_\pi )$: the frequency $\rm{ec}_{\gamma } ({{\mathbb {C}}})$ of edge-configuration $\gamma$ in the set $E^{\rm int}({{\mathbb {C}}})$ of interior-edges in ${{\mathbb {C}}}$.
11. ${\rm dcp}_i({{\mathbb {C}}})$, $i= 14+|\Lambda ^{\rm int}(D_\pi )|+|\Lambda ^{\rm ex}(D_\pi )| + |\Gamma ^{\rm int}(D_\pi )|+ [\psi ]$, $\psi \in {\mathcal {F}}(D_\pi )$: the frequency $\rm{fc}_{\psi }({{\mathbb {C}}})$ of fringe-configuration $\psi$ in the set of ${\rho }$-fringe-trees in ${{\mathbb {C}}}$.
12. ${\rm dcp}_i({{\mathbb {C}}})$, $i= 14+|\Lambda ^{\rm int}(D_\pi )|+|\Lambda ^{\rm ex}(D_\pi )| + |\Gamma ^{\rm int}(D_\pi )|+|{\mathcal {F}}(D_\pi )|+ [\nu ]$, $\nu \in \Gamma _{\rm ac}^{\rm lf}$: the frequency ${\rm ac}_{\nu }^{\rm lf}({{\mathbb {C}}})$ of adjacency-configuration $\nu$ in the set of leaf-edges in $\langle {{\mathbb {C}}}\rangle$.

## Appendix B MILP formulation for the 2L$+$CC Model

Let ${{\mathcal {T}}}=(T;V^{\circ },E^{\circ })$ denote a seed tree. Each ring node $u\in V^{\circ }$ is expanded to a cycle whose length is between $c_{\min }$ and $c_{\max }$. This expansion is done by assigning $\xi \in \Xi$ to *u*, where $\Xi ^u$ is the set of cycle-configurations available to *u* such that every $\xi \in \Xi$ satisfies $c_{\min }\le |\xi |\le c_{\max }$. Strictly speaking, a ring node $u\in V^{\circ }$ is assigned a graph $C^u$ such that $V(C^u)=\{u_1,u_2,\dots ,u_{c_{\max }}\}$ and $E(C^u)=\{e^u_1,e^u_2,\dots ,e^u_{2c_{\max }-c_{\min }}\}$, where, for $i\in [1,2c_{\max }-c_{\min }]$,

$$\begin{aligned} e^u_i=\left\{ \begin{array}{ll} u_iu_{i+1} & \text {if }i\le c_{\max },\\ u_1u_{i-c_{\max }+c_{\min }-1} & \text {otherwise}, \end{array} \right. \end{aligned}$$

and we regard $u_{c_{\max }+1}=u_1$ for convenience. The vertices and edges that form the cycle are chosen according to $|\xi |$, where $\xi$ is the cycle-configuration assigned to *u*. Specifically, vertices $u_1,u_2,\dots ,u_{|\xi |}$ and edges $u_1u_2,\dots ,u_{|\xi |-1}u_{|\xi |},u_{|\xi |}u_1$ are chosen.

For a cycle-configuration $\xi$ and $r\in [1,c_{\max }]$, let us define

$$\begin{aligned} {\hat{\xi }}^{+}(r)\triangleq \{(\mu ,\mu _0)\mid \mu ,\mu _0\in \{u_1,\dots ,u_{|\xi |}\},\ \xi (\mu -\mu _0+1)=r\};\\ {\hat{\xi }}^{-}(r)\triangleq \{(\mu ,\mu _0)\mid \mu ,\mu _0\in \{u_1,\dots ,u_{|\xi |}\},\ \xi (\mu _0-\mu +1)=r\}, \end{aligned}$$

and for $\mu \in V(C^u)$ and $\delta \in \{+,-\}$, we let

$$\begin{aligned} {\hat{\xi }}^{\delta }(r,\mu ):=\{\mu _0\in V(C^u)\mid (\mu ,\mu _0)\in {\hat{\xi }}^{\delta }(r)\}. \end{aligned}$$

### B.1 assigning cycle-configurations to ring nodes

**Constants**

- A seed tree $${{\mathcal {T}}}=(T;V^{\circ },E^{\circ })$$;
- The set $\Xi ^u$ of available cycle-configurations for each $u\in V^{\circ }$, $\Xi ^{{\mathcal {T}}}:= \bigcup _{u \in V^{\circ }} \Xi ^u$;
- A positive constant $\varepsilon _1\in {\mathbb {R}}_+$ that represents a sufficiently small number;
- A positive constant $M_1\in {\mathbb {R}}_+$ that represents a sufficiently large number.

**Variables**

- Real variables $y^u_{[\mu ]}$, $u\in V^{\circ }$, $\mu \in V(C^u)$ that store the mass sum in the fringe-tree attached to vertex $\mu \in V(C^u)$;
- Real variables $z^u_r$, $u\in V^{\circ }$, $r\in [1,c_{\max }]$ that represent the *r*-th smallest mass sum of a fringe-tree in $C^u$;
- Binary variables $x^u_{[\xi ],[\mu _0],\delta }$, $u\in V^{\circ }$, $\xi \in \Xi ^u$, $\mu _0\in \{u_1,\dots ,u_{|\xi |}\}$ and $\delta \in \{+,-\}$, indicating whether $\xi$ is assigned to the starting point $\mu _0$ in the direction $\delta$;
- Binary variables $x_{[\xi ]}^u$, $u \in V^{\circ }$, $\xi \in \Xi ^u$, indicating whether $\xi$ is assigned to $C^u$;
- Integer variables ${\rm{cc}}([\xi ])$, $\xi \in \Xi ^{{\mathcal {T}}}$, cycle-configurations.

**Constraints**

For each $$u\in V^{\circ }$$:

B1 $$\begin{aligned} 0\le {z}^{u}_1,\dots ,z^{u}_{c_{\max }}\le M_1, \end{aligned}$$

B2 $$\begin{aligned}&z^u_r+\varepsilon _1\le z^u_{r+1}, & r\in [1,c_{\max }-1];\end{aligned}$$

B3 $$\begin{aligned}&x_{[\xi ]}^u = \sum _{\mu _0\in \{u_1,\dots ,u_{|\xi |}\}} \sum _{\delta \in \{+,-\}} x_{[\xi ],[\mu _0],\delta }^u, & \xi \in \Xi ^u;\end{aligned}$$

B4 $$\begin{aligned}&\sum _{\xi \in \Xi ^u}x_{[\xi ]}^u=1;\end{aligned}$$

B5 $$\begin{aligned}&y^u_{[\mu ]}\le z^u_r+M_1\big (1-\sum _{\xi \in \Xi ^u}\sum _{\delta \in \{+,-\}}\sum _{\mu _0\in {\hat{\xi }}^\delta (r,\mu )} x^u_{[\xi ],[\mu _0],\delta }\big ), & r\in [1,c_{\max }],\ \mu \in V(C^u);\end{aligned}$$

B6 $$\begin{aligned}&y^u_{[\mu ]}\ge z^u_r-M_1\big (1-\sum _{\xi \in \Xi ^u}\sum _{\delta \in \{+,-\}}\sum _{\mu _0\in {\hat{\xi }}^\delta (r,\mu )} x^u_{[\xi ],[\mu _0],\delta }\big ), & r\in [1,c_{\max }],\ \mu \in V(C^u). \end{aligned}$$

For each $$\xi \in \Xi ^{{\mathcal {T}}}$$:

B7 $$\begin{aligned}&{\rm{cc}}([\xi ]) = \sum _{u \in V^{\circ }, \xi \in \Xi ^u} x_{[\xi ]}^u , & \end{aligned}$$

### B.2 associating ring nodes with ring edges

**Variables**

- Binary variables $e_i^u, u \in V^{\circ }, i \in [1, 2c_{\max }-c_{\min }]$, indicating whether the edge $e_i$ in $C^u$ is used;
- Binary variables $$x^{{\rm edge},u}_{[e],[\nu ]}$$, $$u\in V^{\circ }$$, $$e\in E^{\circ }(u)$$, $$\nu \in E(C^u)$$;
- Binary variables $$x^{{\rm node},u}_{[e'],[\mu ]}$$, $$u\in V^{\circ }$$, $$e'\in {\bar{E}}^{\circ }(u)$$, $$\mu \in V(C^u)$$;

**Constraints**

For each $$u\in V^{\circ }$$:

B8 $$\begin{aligned}&\sum _{\nu \in E(C^u)} x^{{\rm edge}, u}_{[e],[\nu ]}=1, & e\in E^{\circ }(u); \end{aligned}$$

B9 $$\sum\limits_{{\mu \in V(C^{u} )}} {x_{{[e^{\prime}],[\mu ]}}^{{\rm node}, u} } = 1,\quad e^{\prime} \in \bar{E}^{\circ } (u);$$

B10 $$\begin{aligned}&\sum _{e \in E^{\circ }(u)} \sum _{\nu \in (E(C^u))(\mu )} x_{[e], [\nu ]}^{{\rm edge}, u} \le 1, & \mu \in V(C^u); \end{aligned}$$

B11 $$\begin{aligned}&\sum _{e \in E^{\circ }(u)} x_{[e], [\nu ]}^{{\rm edge}, u} \le 1, & \nu \in E(C^u); \end{aligned}$$

For each $$u \in V^{\circ }$$:

B12 $$\begin{gathered} e_{i}^{u} = 1,\quad i \in [1,c_{{\min }} - 1]; \hfill \\ e_{i}^{u} = \sum\limits_{{\xi \in \Xi ^{u},|\xi | > i}} {x_{{[\xi ]}}^{u} ,} \quad i \in [c_{{\min }} ,c_{{\max }} - 2]; \hfill \\ e_{i}^{u} = \sum\limits_{{\xi \in \Xi ^{u} ,|\xi | = c_{{\max }} }} {x_{{[\xi ]}}^{u} } ,\quad i \in \{ c_{{\max }} - 1,c_{{\max }} \} ; \hfill \\ e_{i}^{u} = \sum\limits_{{\xi \in \Xi ^{u} ,|\xi | = i - c_{{\max }} + c_{{\min }} - 1}} {x_{{[\xi ]}}^{u} } ,\quad i \in [c_{{\max }} + 1,2c_{{\max }} - c_{{\min }} ]; \hfill \\ \end{gathered}$$

B13 $$\begin{aligned}&x_{[e], i}^{{\rm edge}, u} \le e_i^u, \qquad \qquad \qquad e \in E^{\circ }(u), i \in [1, 2c_{\max }-c_{\min }]; \end{aligned}$$

B14 $$x_{{[e^{\prime}],i}}^{{\rm node},u} \le \sum\limits_{{\xi \in \Xi ^{u} ,|\xi | \ge i}} {x_{{[\xi ]}}^{u} } ,\quad e^{\prime} \in \bar{E}^{\circ } (u),i \in [c_{{\min }} + 1,c_{{\max }} ];{\text{ }}$$

### B.3 constraints for including fringe-trees

For a leaf-edge $uv \in E(G_{{\mathcal {T}}})$ with $\deg _{G_{{\mathcal {T}}}}(u)=1$, we define the *adjacency-configuration* of *uv* to be an ordered tuple $(\alpha (u),\alpha (v), \beta (uv))$.

**Constants**

- The set ${{\mathcal {F}}}^u$ of the available fringe-trees for each $u \in V(T)$, ${{\mathcal {F}}}^{{\mathcal {T}}}:= \bigcup _{u \in V(T)} {{\mathcal {F}}}^u$;
- The set ${\Gamma _{\rm ac}^{\rm{lf}}}$ of available adjacency-configuration on the set of leaf-edges;
- Functions ${\rm{ms}_{\mathcal {F}}}(\psi ), {\rm{ht}_{\mathcal {F}}}(\psi ), {n_{{\overline{{\mathtt H}}}}}(\psi ), {\rm{ac}_\gamma ^{\rm{lf}}}(\psi )$ denoting the mass, height, number of non-hydrogen non-root atoms, number of leaf-edge adjacency-configurations $\gamma$ of the fringe-tree $\psi$, respectively;
- Integers ${n_{\rm{LB}}}, {n_{\rm{UB}}}$, that represent the lower and upper bounds on the number of non-hydrogen atoms in $G_{{\mathcal {T}}}$, respectively;
- Integers ${n^{\rm{int}}_{\rm{LB}}}, {n^{\rm{int}}_{\rm{UB}}}$, that represent the lower and upper bounds on the number of non-hydrogen atoms in the interior part of $G_{{\mathcal {T}}}$, respectively;
- Integers ${\rm{fc}_{\rm{LB}}}(\psi ), {\rm{fc}_{\rm{UB}}}(\psi ) \in [0, {n_{\rm{UB}}}], \psi \in {{\mathcal {F}}}^{{\mathcal {T}}}$, that represent the lower and upper bounds on the fringe-configurations, respectively;
- Integers ${{\rm ac}^{{\rm lf}}_{{\rm LB}}}(\gamma ), {{\rm ac}^{\rm{lf}}_{{\rm UB}}}(\gamma ) \in [0, {n_{{\rm UB}}}], \gamma \in {\Gamma _{\rm ac}^{{\rm lf}}}$, that represent the lower and upper bounds on the adjacency-configurations of leaf-edges, respectively;

**Variables**

- Binary variables $\delta _{{\mathcal {F}}}(u, [\mu ];[\psi ])$, $u \in V^{\circ }, \mu \in V(C^u)$, $\psi \in {{\mathcal {F}}}^u$, indicating whether the fringe-tree $\psi$ is attached to vertex $\mu \in V(C^u)$;
- Binary variables $\delta _{{\mathcal {F}}}(v;[\psi ])$, $v \in V(T) {\setminus } V^{\circ }$, $\psi \in {{\mathcal {F}}}^v$, indicating whether the fringe-tree $\psi$ is attached to node $v \in V(T) {\setminus } V^{\circ }$;
- Integer variables ${\rm{fc}}([e];[\psi ]) \in [0,2]$, $e \in E^{\circ }$, $\psi \in {{\mathcal {F}}}^{{\mathcal {T}}}$, that stores the number of the fringe-tree $\psi$ is used in the ring edge $e \in E^{\circ }$;
- Integer variable $\rm{rank}$ that represents the rank of $G_{{\mathcal {T}}}$;
- Integer variables $n_G \in [{n_{{\rm LB}}}, {n_{{\rm UB}}}], {n_{\rm int}}\in [{n^{{\rm int}}_{{\rm LB}}}, {n^{{\rm int}}_{{\rm UB}}}]$ that represents the number of non-hydrogen atoms in $G_{{\mathcal {T}}}$ and the interior part of $G_{{\mathcal {T}}}$, respectively;
- Integer variables $\rm{fc}([\psi ]) \in [{\rm{fc}_{\rm{LB}}}(\psi ), {\rm{fc}_{\rm{UB}}}(\psi )]$, $\psi \in {{\mathcal {F}}}^{{\mathcal {T}}}$ that stores the fringe-configurations;
- Integer variables ${\rm{ac}^{{\rm lf}}}([\gamma ]) \in [{{\rm ac}^{\rm{lf}}_{{\rm LB}}}(\gamma ), {{\rm ac}^{{\rm lf}}_{{\rm UB}}}(\gamma )]$, $\gamma \in {\Gamma _{ac}^{{lf}}}$, that stores the adjacency-configurations of leaf-edges;

**Constraints**

For each $$u \in V^{\circ }, \mu \in V(C^u)$$:

B15 $$\begin{aligned}&\sum _{\psi \in {{\mathcal {F}}}^u} \delta _{{\mathcal {F}}}(u, [\mu ]; [\psi ]) = \sum _{\xi \in \Xi ^u, |\xi | \ge [\mu ]} x_{[\xi ]}^u; & \end{aligned}$$

B16 $$\begin{aligned}&\sum _{\psi \in {{\mathcal {F}}}^u} {\rm{ms}_{\mathcal {F}}}(\psi ) \cdot \delta _{{\mathcal {F}}}(u, [\mu ]; [\psi ]) = y_{[\mu ]}^u; & \end{aligned}$$

For each $$v \in V(T) \setminus V^{\circ }$$:

B17 $$\begin{aligned}&\sum _{\psi \in {{\mathcal {F}}}^v} \delta _{{\mathcal {F}}}(v; [\psi ]) = 1, & \nonumber \\&\sum _{\psi \in {{\mathcal {F}}}^v, {\rm{ht}_{\mathcal {F}}}(\psi ) = \rho } \delta _{{\mathcal {F}}}(v; [\psi ]) = 1, & v \ \text {is a leaf of} \ T; \end{aligned}$$

For each $e=uu'\in E^{\circ }$ such that $[u]<[u']$:

B18 $$\begin{gathered} \sum _{{\psi \in {\mathcal{F}}^{u} }} [\psi ] \cdot \delta _{{\mathcal{F}}} (u,i_{1} ;[\psi ]) - \sum\limits_{{\psi \in {\mathcal{F}}^{{u^{\prime}}} }} {[\psi ] \cdot \delta _{{\mathcal{F}}} } (u^{\prime},j_{2} ;[\psi ]) \le \left| {F^{{\mathcal{T}}} } \right|\left( {2 - x_{{[e],[\nu ]}}^{{\rm edge},u} - x_{{[e],[\nu ^{\prime}]}}^{{\rm edge},u^{\prime}} } \right) \hfill \\ \sum _{{\psi \in {\mathcal{F}}^{u} }} [\psi ] \cdot \delta _{{\mathcal{F}}} (u,i_{1} ;[\psi ]) - \sum\limits_{{\psi \in {\mathcal{F}}^{{u^{\prime}}} }} {[\psi ] \cdot \delta _{{\mathcal{F}}} } (u^{\prime},j_{2} ;[\psi ]) \ge \left| {F^{{\mathcal{T}}} } \right|\left( {x_{{[e],[\nu ]}}^{{\rm edge},u} + x_{{[e],[\nu ^{\prime}]}}^{{\rm edge},u^{\prime}} - 2} \right) \hfill \\ \sum _{{\psi \in {\mathcal{F}}^{u} }} [\psi ] \cdot \delta _{{\mathcal{F}}} (u,i_{2} ;[\psi ]) - \sum\limits_{{\psi \in {\mathcal{F}}^{{u^{\prime}}} }} {[\psi ] \cdot \delta _{{\mathcal{F}}} } (u^{\prime},j_{1} ;[\psi ]) \le \left| {F^{{\mathcal{T}}} } \right|\left( {2 - x_{{[e],[\nu ]}}^{{\rm edge},u} - x_{{[e],[\nu ^{\prime}]}}^{{\rm edge},u^{\prime}} } \right) \hfill \\ \sum _{{\psi \in {\mathcal{F}}^{u} }} [\psi ] \cdot \delta _{{\mathcal{F}}} (u,i_{2} ;[\psi ]) - \sum\limits_{{\psi \in {\mathcal{F}}^{{u^{\prime}}} }} {[\psi ] \cdot \delta _{{\mathcal{F}}} } (u^{\prime},j_{1} ;[\psi ]) \ge \left| {F^{{\mathcal{T}}} } \right|\left( {x_{{[e],[\nu ]}}^{{\rm edge},u} + x_{{[e],[\nu ^{\prime}]}}^{{\rm edge},u^{\prime}} - 2} \right) \hfill \\ \nu = u_{{i_{1} }} u_{{i_{2} }} \in E(C^{u} ),\nu ^{\prime} = u^{\prime}_{{j_{1} }} u^{\prime}_{{j_{2} }} \in E(C^{{u^{\prime}}} ) \hfill \\ {\text{such that }}i_{1} < i_{2} ,j < j_{2} ; \hfill \\ \end{gathered}$$

B19 $$\begin{aligned} {\rm fc}([e]; [\psi ]) - \delta _{{\mathcal {F}}}(u, i_1; [\psi ]) - \delta _{{\mathcal {F}}}(u, i_2; [\psi ]) \le 2(1 - x_{[e], [\nu ]}^{{\rm edge}, u}),&\nonumber \\ {\rm fc}([e]; [\psi ]) - \delta _{{\mathcal {F}}}(u, i_1; [\psi ]) - \delta _{{\mathcal {F}}}(u, i_2; [\psi ]) \ge 2( x_{[e], [\nu ]}^{{\rm edge}, u} - 1),&\nu = u_{i_1}u_{i_2} \in E(C^u), \psi \in {{\mathcal {F}}}^{{\mathcal {T}}}; \end{aligned}$$

For each $$\psi \in {{\mathcal {F}}}^{{\mathcal {T}}}$$:

B20 $$\begin{aligned}&{\rm fc}([\psi ]) = \sum _{u \in V^{\circ }} \sum _{\mu \in V(C^u)} \delta _{{\mathcal {F}}}(u, [\mu ]; [\psi ]) + \sum _{v \in V(T) \setminus V^{\circ }} \delta _{{\mathcal {F}}}(v; [\psi ]) - \sum _{e \in E^{\circ }} {\rm fc}([e];[\psi ]); & \end{aligned}$$

For each $$\gamma \in {\Gamma _{ac}^{{lf}}}$$:

B21 $$\begin{aligned}&{\rm{ac}^{\rm{lf}}}([\gamma ]) = \sum _{\psi \in {{\mathcal {F}}}^{{\mathcal {T}}}} {\rm{ac}_\gamma ^{\rm{lf}}}(\psi ) \rm{fc}([\psi ]); \end{aligned}$$

B22 $$\begin{aligned}&{\rm rank}= |V^{\circ }|; & \end{aligned}$$

B23 $$\begin{aligned}&{n_{\rm int}}= \sum _{u \in V^{\circ }} \sum _{\xi \in \Xi ^u} |\xi | \cdot x_{[\xi ]}^u + |V(T) \setminus V^{\circ }| - 2|E^{\circ }|; & \end{aligned}$$

B24 $$\begin{aligned}&n_G = {n_{\rm int}}+ \sum _{\psi \in {{\mathcal {F}}}^{{\mathcal {T}}}} {n_{{\overline{{\mathtt H}}}}}(\psi ){\rm fc}([\psi ]); & \end{aligned}$$

### B.4 descriptors for the number of specified degree

**Constants**

- Function ${\rm{deg}_{{\overline{{\mathtt H}}}}}(\psi )$ denoting the degree of the root of the fringe tree $\psi$;

**Variables**

- Binary variables ${\delta _{\rm{deg}}}(u, [\mu ]; d), u \in V^{\circ }, \mu \in V(C^u), d \in [1,4]$, indicating the degree of $\mu$ in $G_{{{\mathcal {T}}}}$;
- Binary variables ${\delta _{\rm{deg}}}(v; d), v \in V(T) \setminus V^{\circ }, d \in [1,4]$, indicating the degree of *v* in $G_{{{\mathcal {T}}}}$;
- Binary variables ${\delta ^{\rm{int}}_{\rm{deg}}}(u, [\mu ]; d), u \in V^{\circ }, \mu \in V(C^u), d \in [1,4]$, indicating the interior degree of $\mu$ in $G_{{{\mathcal {T}}}}$;
- Binary variables ${\delta ^{\rm{int}}_{\rm{deg}}}(v; d), v \in V(T) \setminus V^{\circ }, d \in [1,4]$, indicating the interior degree of *v* in $G_{{{\mathcal {T}}}}$;
- Integer variables ${\rm dg}(d), d \in [1,4]$, that stores the number of vertices with degree *d* in $G_{{\mathcal {T}}}$;
- Integer variables ${\rm{deg}^{\rm{int}}}(d), d \in [1,4]$, that stores the number of vertices with interior degree *d* in $G_{{\mathcal {T}}}$;
- Integer variables ${\rm dg}([e];d) \in [0,2], e \in E^{\circ }, d \in [1,4]$, that stores the number of vertices with degree *d* in the ring edge *e*;
- Integer variables ${\rm{deg}^{\rm{int}}}([e];d) \in [0,2], e \in E^{\circ }, d \in [1,4]$, that stores the number of vertices with interior degree *d* in the ring edge *e*;
- Integer variables ${\rm dg}_{[e], [\mu ], +}^{{\rm edge}, u}, u \in V^{\circ }, \mu \in V(C^u), e \in E^{\circ }(u)$, indicating the degree other than that of the ring edge *e* at $\mu$ in $C^u$;
- Integer variables ${\rm dg}_{[e], [\mu ], -}^{{\rm edge}, u}, u \in V^{\circ }, \mu \in V(C^u), e \in E^{\circ }(u)$, indicating the augmented degree for $\mu$ because of the ring edge *e*;

**Constraints**

For each $$u \in V^{\circ }, \mu \in V(C^u)$$:

B25 $$\begin{aligned}&0 \le {\rm dg}_{[e], [\mu ], +}^{{\rm edge}, u} \le 4 \sum _{\nu \in (E(C^u))(\mu )} x_{[e], [\nu ]}^{{\rm edge}, u} & e \in E^{\circ }(u); \nonumber \\&0 \le {\rm dg}_{[e], [\mu ], -}^{{\rm edge}, u} \le 4 \sum _{\nu \in (E(C^u))(\mu )} x_{[e], [\nu ]}^{{\rm edge}, u} & e \in E^{\circ }(u); \end{aligned}$$

B26 $$\begin{aligned}&4(x_{[e],[\nu ]}^{{\rm edge}, u} - 1) \le {\rm dg}_{[e],[\mu ], +}^{{\rm edge}, u} -1 - \sum _{e' \in {\bar{E}}^{\circ }(u)} x_{[e'], [\mu ]}^{{\rm node}, u} \le 4(1 - x_{[e],[\nu ]}^{{\rm edge}, u}), & e \in E^{\circ }(u), \nu \in (E(C^u))(\mu ); \end{aligned}$$

For each $e=uu'\in E^{\circ }$ such that $[u]<[u']$:

B27 $$\begin{aligned} 3 (x_{[e],[\nu ]}^{{\rm edge}, u} + x_{[e],[\nu ']}^{{\rm edge}, u'} - 2) \le {\rm dg}_{[e], i_1, -}^{{\rm edge}, u} - {\rm dg}_{[e],j_2, +}^{{\rm edge}, u'} \le 3 (2-x_{[e],[\nu ]}^{{\rm edge}, u} - x_{[e],[\nu ']}^{{\rm edge}, u'}),&\nonumber \\ 3 (x_{[e],[\nu ]}^{{\rm edge}, u} + x_{[e],[\nu ']}^{{\rm edge}, u'} - 2) \le {\rm dg}_{[e], i_2, -}^{{\rm edge}, u} - {\rm dg}_{[e],j_1, +}^{{\rm edge}, u'} \le 3 (2-x_{[e],[\nu ]}^{{\rm edge}, u} - x_{[e],[\nu ']}^{{\rm edge}, u'}),&\nonumber \\ 3 (x_{[e],[\nu ]}^{{\rm edge}, u} + x_{[e],[\nu ']}^{{\rm edge}, u'} - 2) \le {\rm dg}_{[e], j_2, -}^{{\rm edge}, u'} - {\rm dg}_{[e],i_1, +}^{{\rm edge}, u} \le 3 (2-x_{[e],[\nu ]}^{{\rm edge}, u} - x_{[e],[\nu ']}^{{\rm edge}, u'}),&\nonumber \\ 3 (x_{[e],[\nu ]}^{{\rm edge}, u} + x_{[e],[\nu ']}^{{\rm edge}, u'} - 2) \le {\rm dg}_{[e], j_1, -}^{{\rm edge}, u'} - {\rm dg}_{[e],i_2, +}^{{\rm edge}, u} \le 3 (2-x_{[e],[\nu ]}^{{\rm edge}, u} - x_{[e],[\nu ']}^{{\rm edge}, u'}),&\nonumber \\ \nu =u_{i_1}u_{i_2}\in E(C^u),\ \nu '=u'_{j_1}u'_{j_2}\in E(C^{u'})&\nonumber \\ \text {such that }i_1<i_2,\ j_1<j_2;&\end{aligned}$$

For each $$u \in V^{\circ }, \mu \in V(C^u)$$:

B28 $$\begin{aligned} \sum _{d \in [1,4]} {\delta _{\rm{deg}}}(u, [\mu ]; d)&= \sum _{\xi \in \Xi ^u, |\xi | \ge [\mu ]} x_{[\xi ]}^u; \end{aligned}$$

B29 $$\begin{aligned} 2(\sum _{\xi \in \Xi ^u, |\xi | \ge [\mu ]} x_{[\xi ]}^u - 1)&\le \sum _{d \in [1,4]} d \cdot {\delta _{{\rm deg}}}(u, [\mu ]; d) - (2 + \sum _{\psi \in {{\mathcal {F}}}^u} {{\rm deg}_{{\overline{{\mathtt H}}}}}(\psi ) \delta _{{\mathcal {F}}}(u, [\mu ]; [\psi ]) + \sum _{e' \in {\bar{E}}^{\circ }(u)} x_{[e'], [\mu ]}^{{\rm node}, u} +\sum _{e\in E^{\circ }(u)} {\rm dg}_{[e], [\mu ], -}^{{\rm edge}, u}); \nonumber \\ \sum _{d \in [1,4]} d \cdot {\delta _{{\rm deg}}}(u, [\mu ]; d)&\le \ 2 + \sum _{\psi \in {{\mathcal {F}}}^u} {{\rm deg}_{{\overline{{\mathtt H}}}}}(\psi ) \delta _{{\mathcal {F}}}(u, [\mu ]; [\psi ]) + \sum _{e' \in {\bar{E}}^{\circ }(u)} x_{[e'], [\mu ]}^{{\rm node}, u} +\sum _{e\in E^{\circ }(u)} {\rm dg}_{[e], [\mu ], -}^{{\rm edge}, u}); \end{aligned}$$

B30 $$\begin{aligned} \sum _{d \in [1,4]} {\delta ^{\rm{int}}_{\rm{deg}}}(u, [\mu ]; d)&= \sum _{\xi \in \Xi ^u, |\xi | \ge [\mu ]} x_{[\xi ]}^u; \end{aligned}$$

B31 $$\begin{aligned} 2(\sum _{\xi \in \Xi ^u, |\xi | \ge [\mu ]} x_{[\xi ]}^u - 1)&\le \sum _{d \in [1,4]} d \cdot {\delta ^{{\rm int}}_{{\rm deg}}}(u, [\mu ]; d) - \ (2 + \sum _{e' \in {\bar{E}}^{\circ }(u)} x_{[e'], [\mu ]}^{{\rm node}, u} + \sum _{e\in E^{\circ }(u)} {\rm dg}_{[e], [\mu ], -}^{{\rm edge}, u}); \nonumber \\ \sum _{d \in [1,4]} d \cdot {\delta ^{{\rm int}}_{{\rm deg}}}(u, [\mu ]; d)&\le \ 2 + \sum _{e' \in {\bar{E}}^{\circ }(u)} x_{[e'], [\mu ]}^{{\rm node}, u} + \sum _{e\in E^{\circ }(u)} {\rm dg}_{[e], [\mu ], -}^{{\rm edge}, u}); \end{aligned}$$

For each $$v \in V(T) \setminus V^{\circ }$$:

B32 $$\begin{aligned}&\sum _{d \in [1,4]} {\delta _{\rm{deg}}}(v; d) = 1; \end{aligned}$$

B33 $$\begin{aligned}&\sum _{d \in [1,4]} d \cdot {\delta _{\rm{deg}}}(v; d) = |N_T(v)| + \sum _{\psi \in {{\mathcal {F}}}^v} {\rm{deg}_{{\overline{{\mathtt H}}}}}(\psi ) \delta _{{\mathcal {F}}}(v; [\psi ]); \end{aligned}$$

B34 $$\begin{aligned}&\sum _{d \in [1,4]} {\delta ^{{\rm int}}_{{\rm deg}}}(v; d) = 1; \end{aligned}$$

B35 $$\begin{aligned}&\sum _{d \in [1,4]} d \cdot {\delta ^{{\rm int}}_{{\rm deg}}}(v; d) = |N_T(v)|; \end{aligned}$$

For each $e=uu'\in E^{\circ }$ such that $[u]<[u']$:

B36 $$\begin{aligned} {\rm dg}([e]; d) - {\delta _{{\rm deg}}}(u, i_1; d) - {\delta _{{\rm deg}}}(u, i_2; d) \le 2(1 - x_{[e], [\nu ]}^{{\rm edge}, u}),&\nonumber \\ {\rm dg}([e]; d) - {\delta _{{\rm deg}}}(u, i_1; d) - {\delta _{{\rm deg}}}(u, i_2; d) \ge 2(x_{[e], [\nu ]}^{{\rm edge}, u} - 1),&\nu = u_{i_1}u_{i_2}\in E(C^u), d \in [1,4] \end{aligned}$$

B37 $$\begin{aligned} {{\rm deg}^{{\rm int}}}([e]; d) - {\delta ^{{\rm int}}_{{\rm deg}}}(u, i_1; d) - {\delta ^{{\rm int}}_{{\rm deg}}}(u, i_2; d) \le 2(1- x_{[e], [\nu ]}^{{\rm edge}, u}),&\nonumber \\ {{\rm deg}^{{\rm int}}}([e]; d) - {\delta ^{{\rm int}}_{{\rm deg}}}(u, i_1; d) - {\delta ^{{\rm int}}_{{\rm deg}}}(u, i_2; d) \ge 2( x_{[e], [\nu ]}^{{\rm edge}, u} - 1),&\nu = u_{i_1}u_{i_2}\in E(C^u), d \in [1,4] \end{aligned}$$

For each $$d \in [1,4]$$:

B38 $$\begin{aligned}&{\rm dg}(d) = \sum _{u \in V^{\circ }} \sum _{\mu \in V(C^u)} {\delta _{{\rm deg}}}(u, [\mu ]; d) + \sum _{v \in V(T) \setminus V^{\circ }} {\delta _{{\rm deg}}}(v; d) - \sum _{e \in E^{\circ }} {\rm dg}([e];d); \end{aligned}$$

B39 $$\begin{aligned}&{{\rm deg}^{{\rm int}}}(d) = \sum _{u \in V^{\circ }} \sum _{\mu \in V(C^u)} {\delta ^{{\rm int}}_{{\rm deg}}}(u, [\mu ]; d) + \sum _{v \in V(T) \setminus V^{\circ }} {\delta ^{{\rm int}}_{{\rm deg}}}(v; d) - \sum _{e \in E^{\circ }} {{\rm deg}^{{\rm int}}}([e];d); \end{aligned}$$

### B.5 Assigning bond-multiplicity

**Variables**

- Integer variables $\beta _i^u \in [0,3], u \in V^{\circ }, i \in [1, 2c_{\max }-c_{\min }]$, that stores the bond-multiplicty of the edge $e_i$ in $C^u$;
- Integer variables $\beta _{[e]} \in [1,3], e \in E(T)$, that stores the bond-multiplicty of the edge *e*;
- Binary variables $${\delta _\beta }(u, i; m), u \in V^{\circ }, i \in [1, 2c_{\max }-c_{\min }], m \in [1,3]$$, $${\delta _\beta }(u,i;m)=1 \Leftrightarrow \beta _i^u = m$$;
- Binary variables $${\delta _\beta }([e]; m), e \in E(T), m \in [1,3]$$, $${\delta _\beta }([e];m)=1 \Leftrightarrow \beta _{[e]} = m$$;
- Bnteger variables ${\rm bd}(m), m \in [1,3]$, that stores the number of edges with bond-multiplicty *m*;

**Constraints**

B40 $$\begin{aligned}&e_i^u \le \beta _i^u \le 3 e_i^u, & u \in V^{\circ }, i \in [1, 2c_{\max }-c_{\min }]; \end{aligned}$$

B41 $$\begin{aligned}&1 \le \beta _{[e]} \le 3, & e \in E(T); \end{aligned}$$

B42 $$\begin{aligned}&\sum _{m \in [1,3]} {\delta _\beta }(u, i;m) = e_i^u,&\sum _{m \in [1,3]} m \cdot {\delta _\beta }(u,i;m) = \beta _i^u, & u \in V^{\circ }, i \in [1, 2c_{\max }-c_{\min }]; \end{aligned}$$

B43 $$\begin{aligned}&\sum _{m \in [1,3]} {\delta _\beta }([e]; m) = 1,&\sum _{m \in [1,3]} m \cdot {\delta _\beta }([e]; m) = \beta _{[e]}, & e \in E(T); \end{aligned}$$

For each $$u \in V^{\circ }, e \in E^{\circ }(u)$$:

B44 $$\begin{aligned}&3(x_{[e],i}^{{\rm edge}, u} - 1) \le \beta _i^u - \beta _{[e]} \le 3(1 - x_{[e],i}^{{\rm edge}, u}), & i \in [1, 2c_{\max }-c_{\min }]; \end{aligned}$$

For each $$m \in [1,3]$$:

B45 $${\rm bd}(m) = \sum\limits_{{u \in V^{\circ } }} {\sum\limits_{{i \in [1,2c_{{\max }} - c_{{\min }} ]}} {\delta _{\beta } } } (u,i;m) + \sum\limits_{{e^{\prime} \in E(T){ \setminus }E^{\circ } }} {\delta _{\beta } } ([e^{\prime}];m) - \sum\limits_{{e \in E^{\circ } }} {\delta _{\beta } } ([e];m);$$

### B.6 Assigning chemical elements and valence condition

**Constants**

- A set $\Lambda$ consisting of all available chemical elements;
- Functions ${\alpha _{\rm{r}}}(\psi ), \rm{val}_{\mathcal {F}}^{\rm{ex}}(\psi ), \rm{eledeg}_{\mathcal {F}}(\psi ), {n_{{\mathtt a}}}(\psi )$ denoting the chemical element of the root, root valence, ion-valence, number of non-root chemical element ${\mathtt a}$ of the fringe-tree $\psi$, respectively;
- Functions $\rm{val}({\mathtt a}), \rm{mass}^*({\mathtt a})$ denoting the valence and mass of the chemical element ${\mathtt a}$, respectively;
- Integers ${\rm{na}^{\rm{int}}_{\rm{LB}}}([{\mathtt a}]), {\rm{na}^{\rm{int}}_{\rm{UB}}}([{\mathtt a}]) \in [0, {n_{\rm{UB}}}], {\mathtt a}\in \Lambda$, that represent the lower and upper bounds of the chemical element ${\mathtt a}$ in the interior part, respectively;
- Integers ${\rm{na}^{\rm{ex}}_{\rm{LB}}}([{\mathtt a}]), {\rm{na}^{\rm{ex}}_{\rm{UB}}}([{\mathtt a}]) \in [0, {n_{\rm{UB}}}], {\mathtt a}\in \Lambda$, that represent the lower and upper bounds of the chemical element ${\mathtt a}$ in the exterior part, respectively;
- Integers ${\rm{na}_{\rm{LB}}}([{\mathtt a}]), {\rm{na}_{\rm{UB}}}([{\mathtt a}]) \in [0, {n_{\rm{UB}}}], {\mathtt a}\in \Lambda$, that represent the lower and upper bounds of the chemical element ${\mathtt a}$ in $G_{{\mathcal {T}}}$, respectively;
- A positive constant $M_{\rm{ms}} \in {\mathbb {R}}_+$ that represents a sufficiently large number;

**Variables**

- Integer variables $\alpha (u, [\mu ]), u \in V^{\circ }, \mu \in V(C^u)$, that represents the chemical element assigned to the vertex $\mu$ in $C^u$;
- Integer variables $\alpha (v), v \in V(T) \setminus V^{\circ }$, that represents the chemical element assigned to the vertex *v*;
- Binary variables $${\delta _\alpha }(u, [\mu ]; [{\mathtt a}]), u \in V^{\circ }, \mu \in V(C^u), {\mathtt a}\in \Lambda$$, $${\delta _\alpha }(u,[\mu ]; [{\mathtt a}]) = 1 \Leftrightarrow \alpha (u, [\mu ]) = [{\mathtt a}]$$;
- Binary variables $${\delta _\alpha }(v; [{\mathtt a}]), v \in V(T) {\setminus } V^{\circ }, {\mathtt a}\in \Lambda$$, $${\delta _\alpha }(v; [{\mathtt a}]) = 1 \Leftrightarrow \alpha (v) = [{\mathtt a}]$$;
- Integer variables $\beta _{[e'], [\mu ]}^{{\rm node}, u}, u \in V^{\circ }, \mu \in V(C^u), e' \in {\bar{E}}^{\circ }(u)$, indicating the bond-multiplicity assigned to the non-ring edge $e'$ at vertex $\mu$;
- Integer variables $\beta _{[e], [\mu ], +}^{{\rm edge}, u}, u \in V^{\circ }, \mu \in V(C^u), e \in E^{\circ }(u)$, indicating the bond-multiplicity other than that of the ring edge *e* at $\mu$ in $C^u$;
- Integer variables $\beta _{[e], [\mu ], -}^{{\rm edge}, u}, u \in V^{\circ }, \mu \in V(C^u), e \in E^{\circ }(u)$, indicating the augmented bond-multiplicity for $\mu$ because of the ring edge *e*;
- Integer variables ${\rm na}([e]; [{\mathtt a}])\in [0,2], e \in E^{\circ }, {\mathtt a}\in \Lambda$, that stores the number of chemical element ${\mathtt a}$ used in the ring edge *e*;
- Integer variables ${\rm{na}^{\rm{int}}}([{\mathtt a}]) \in [{\rm{na}^{\rm{int}}_{\rm{LB}}}([{\mathtt a}]), {\rm{na}^{\rm{int}}_{\rm{UB}}}([{\mathtt a}])], {\mathtt a}\in \Lambda$, that stores the number of chemical element ${\mathtt a}$ in the interior part;
- Integer variables ${\rm{na}^{\rm{ex}}}([{\mathtt a}]) \in [{\rm{na}^{\rm{ex}}_{\rm{LB}}}([{\mathtt a}]), {\rm{na}^{\rm{ex}}_{\rm{UB}}}([{\mathtt a}])], {\mathtt a}\in \Lambda$, that stores the number of chemical element ${\mathtt a}$ in the exterior part;
- Integer variables $\rm{na}([{\mathtt a}]) \in [{\rm{na}_{\rm{LB}}}([{\mathtt a}]), {\rm{na}_{\rm{UB}}}([{\mathtt a}])], {\mathtt a}\in \Lambda$, that stores the number of chemical element ${\mathtt a}$ in $G_{{\mathcal {T}}}$;
- Binary variables $${\delta _{\rm{atm}}}(i), i \in [{n_{\rm{LB}}}+{\rm{na}_{\rm{LB}}}([{\mathtt H}]), {n_{\rm{UB}}}+{\rm{na}_{\rm{UB}}}([{\mathtt H}])]$$, $${\delta _{\rm{atm}}}(i) = 1 \Leftrightarrow n_G = i$$;
- Integer variable $\rm{Mass}$ that represents the total mass of $G_{{\mathcal {T}}}$;
- Real variable ${\overline{\rm{ms}}}$ that represents the average mass of $G_{{\mathcal {T}}}$;

**Constraints**

For each $$u \in V^{\circ }, \mu \in V(C^u)$$:

B46 $$\begin{aligned}&\alpha (u, [\mu ]) = \sum _{\psi \in {{\mathcal {F}}}^u} [{\alpha _{\rm{r}}}(\psi )] \cdot \delta _{{\mathcal {F}}}(u, [\mu ]; [\psi ]); & \end{aligned}$$

B47 $$\begin{aligned}&\sum _{{\mathtt a}\in \Lambda } {\delta _\alpha }(u, [\mu ]; [{\mathtt a}]) = \sum _{\xi \in \Xi ^u, |\xi | \ge [\mu ]} x_{[\xi ]}^u; & \end{aligned}$$

B48 $$\begin{aligned}&\sum _{{\mathtt a}\in \Lambda } [{\mathtt a}] \cdot {\delta _\alpha }(u, [\mu ]; [{\mathtt a}]) = \alpha (u,[\mu ]); & \end{aligned}$$

For each $$v \in V(T) \setminus V^{\circ }$$:

B49 $$\begin{aligned}&\alpha (v) = \sum _{\psi \in {{\mathcal {F}}}^v} [{\alpha _{\rm{r}}}(\psi )] \cdot \delta _{{\mathcal {F}}}(v; [\psi ]); & \end{aligned}$$

B50 $$\begin{aligned}&\sum _{{\mathtt a}\in \Lambda } {\delta _\alpha }(v; [{\mathtt a}]) = 1; & \end{aligned}$$

B51 $$\begin{aligned}&\sum _{{\mathtt a}\in \Lambda } [{\mathtt a}] \cdot {\delta _\alpha }(v; [{\mathtt a}]) = \alpha (v); & \end{aligned}$$

For each $$u \in V^{\circ }, \mu \in V(C^u)$$:

B52 $$0 \le \beta _{{[e^{\prime}],[\mu ]}}^{{{\text{node}},{u}}} \le 3x_{{[e^{\prime}],[\mu ]}}^{{\rm node},u} ,\quad e^{\prime} \in \bar{E}^{\circ } (u);$$

B53 $$\begin{aligned}3(x_{[e'],[\mu ]}^{{\rm node}, u} - 1) \le \beta _{[e']} - \beta _{[e'], [\mu ]}^{{\rm node}, u} \le 3(1 - x_{[e'],[\mu ]}^{{\rm node}, u}), \quad e' \in {\bar{E}}^{\circ }(u);\end{aligned}$$

B54 $$\begin{aligned}&0 \le \beta _{[e], [\mu ], +}^{{\rm edge}, u} \le 6 \sum _{\nu \in (E(C^u))(\mu )} x_{[e], [\nu ]}^{{\rm edge}, u} & e \in E^{\circ }(u); \nonumber \\&0 \le \beta _{[e], [\mu ], -}^{{\rm edge}, u} \le 6 \sum _{\nu \in (E(C^u))(\mu )} x_{[e], [\nu ]}^{{\rm edge}, u} & e \in E^{\circ }(u); \end{aligned}$$

B55 $$\begin{aligned}&6(x_{[e],[\nu ]}^{{\rm edge}, u} - 1) \le \beta _{[e],[\mu ], +}^{{\rm edge}, u} + \beta _{[e]} - \sum _{\nu ' \in (E(C^u))(\mu )} \beta _{[\nu ']}^u - \sum _{e' \in {\bar{E}}^{\circ }(u)} \beta _{[e'], [\mu ]}^{{\rm node}, u} \le 6(1 - x_{[e],[\nu ]}^{{\rm edge}, u}), & e \in E^{\circ }(u), \nu \in (E(C^u))(\mu ); \end{aligned}$$

For each $e=uu'\in E^{\circ }$ such that $[u]<[u']$:

B56 $$\begin{aligned} 3 (x_{[e],[\nu ]}^{{\rm edge}, u} + x_{[e],[\nu ']}^{{\rm edge}, u'} - 2) \le \beta _{[e], i_1, -}^{{\rm edge}, u} - \beta _{[e],j_2, +}^{{\rm edge}, u'} \le 3 (2-x_{[e],[\nu ]}^{{\rm edge}, u} - x_{[e],[\nu ']}^{{\rm edge}, u'}), \nonumber \\&3 (x_{[e],[\nu ]}^{{\rm edge}, u} + x_{[e],[\nu ']}^{{\rm edge}, u'} - 2) \le \beta _{[e], i_2, -}^{{\rm edge}, u} - \beta _{[e],j_1, +}^{{\rm edge}, u'} \le 3 (2-x_{[e],[\nu ]}^{{\rm edge}, u} - x_{[e],[\nu ']}^{{\rm edge}, u'}), \nonumber \\&3 (x_{[e],[\nu ]}^{{\rm edge}, u} + x_{[e],[\nu ']}^{{\rm edge}, u'} - 2) \le \beta _{[e], j_2, -}^{{\rm edge}, u'} - \beta _{[e],i_1, +}^{{\rm edge}, u} \le 3 (2-x_{[e],[\nu ]}^{{\rm edge}, u} - x_{[e],[\nu ']}^{{\rm edge}, u'}), \nonumber \\&3 (x_{[e],[\nu ]}^{{\rm edge}, u} + x_{[e],[\nu ']}^{{\rm edge}, u'} - 2) \le \beta _{[e], j_1, -}^{{\rm edge}, u'} - \beta _{[e],i_2, +}^{{\rm edge}, u} \le 3 (2-x_{[e],[\nu ]}^{{\rm edge}, u} - x_{[e],[\nu ']}^{{\rm edge}, u'}), \nonumber \\&\nu =u_{i_1}u_{i_2}\in E(C^u),\ \nu '=u'_{j_1}u'_{j_2}\in E(C^{u'}) \nonumber \\&\text {such that }i_1<i_2,\ j_1<j_2; \end{aligned}$$

B57 $$\begin{aligned}&\sum _{{\mathtt a}\in \Lambda } {\rm val}({\mathtt a}) \cdot {\delta _\alpha }(u, [\mu ];[{\mathtt a}]) &= \sum _{\nu \in (E(C^u))(\mu )} \beta _{[\nu ]}^u + \sum _{e' \in {\bar{E}}^{\circ }(u)} \beta _{[e'], [\mu ]}^{{\rm node}, u} + \sum _{e \in E^{\circ }(u)} \beta _{[e],[\mu ],-}^{{\rm edge}, u} \nonumber \\ && + \sum _{\psi \in {{\mathcal {F}}}^u} ({\rm val}_{\mathcal {F}}^{{\rm ex}}(\psi ) - {\rm eledeg}_{\mathcal {F}}(\psi )) \delta _{{\mathcal {F}}}(u, [\mu ];[\psi ]),&u \in V^{\circ }, \mu \in V(C^u); \end{aligned}$$

B58 $$\begin{aligned}&\sum _{{\mathtt a}\in \Lambda } {\rm val}({\mathtt a}) \cdot {\delta _\alpha }(v;[{\mathtt a}]) = \sum _{e' \in {\bar{E}}^{\circ }(v)} \beta _{[e']} + \sum _{\psi \in {{\mathcal {F}}}^v} ({\rm val}_{\mathcal {F}}^{{\rm ex}}(\psi ) - {\rm eledeg}_{\mathcal {F}}(\psi )) \delta _{{\mathcal {F}}}(v;[\psi ]),&v \in V(T) \setminus V^{\circ }; \end{aligned}$$

For each $${\mathtt a}\in \Lambda$$:

B59 $$\begin{aligned}&{\rm na}([e];[{\mathtt a}]) = \sum _{\psi \in {{\mathcal {F}}}^{{\mathcal {T}}}, {\alpha _{\rm{r}}}(\psi ) = {\mathtt a}} {\rm fc}([e]; [\psi ]),&e \in E^{\circ }; \end{aligned}$$

B60 $$\begin{aligned}&{\rm{na}^{\rm{int}}}([{\mathtt a}]) = \sum _{u \in V^{\circ }} \sum _{\mu \in V(C^u)} {\delta _\alpha }(u, [\mu ];[{\mathtt a}]) + \sum _{v \in V(T) \setminus V^{\circ }} {\delta _\alpha }(v;[{\mathtt a}]) - \sum _{e \in E^{\circ }} {\rm na}([e]; [{\mathtt a}]); \end{aligned}$$

B61 $$\begin{aligned}&{\rm{na}^{\rm{ex}}}([{\mathtt a}]) = \sum _{\psi \in {{\mathcal {F}}}^{{\mathcal {T}}}} {n_{{\mathtt a}}}(\psi ) \cdot \rm{fc}([\psi ]); \end{aligned}$$

B62 $$\begin{aligned}&\rm{na}([{\mathtt a}]) = {\rm{na}^{\rm{int}}}([{\mathtt a}]) + {\rm{na}^{\rm{ex}}}([{\mathtt a}]); \end{aligned}$$

B63 $$\begin{aligned}&\rm{Mass}= \sum _{{\mathtt a}\in \Lambda } \rm{mass}^*({\mathtt a}) \cdot \rm{na}([{\mathtt a}]); \end{aligned}$$

B64 $$\begin{aligned}&\sum _{i \in [{n_{{\rm LB}}}+ {{\rm na}_{{\rm LB}}}([{\mathtt H}]), {n_{{\rm UB}}}+{{\rm na}_{{\rm UB}}}([{\mathtt H}])]} {\delta _{{\rm atm}}}(i) = 1; \end{aligned}$$

B65 $$\begin{aligned}&\sum _{i \in [{n_{{\rm LB}}}+ {{\rm na}_{{\rm LB}}}([{\mathtt H}]), {n_{{\rm UB}}}+{{\rm na}_{{\rm UB}}}([{\mathtt H}])]} i \cdot {\delta _{{\rm atm}}}(i) = n_G + {{\rm na}^{{\rm ex}}}([{\mathtt H}]); \end{aligned}$$

B66 $$M_{{\rm ms}} (\delta _{{\rm atm}} (i) - 1) \le \overline{{\rm ms}} - \frac{{\rm Mass}}{i} \le M_{{\rm ms}} (1 - \delta _{{\rm atm}} (i)),i \in [n_{{\rm LB}} + na_{{\rm LB}} ([{\mathtt H}]),n_{{\rm UB}} + na_{{\rm UB}} ([{\mathtt H}])];$$

### B.7 Descriptors for the number of adjacency-configurations

**Constants**

- A set ${\Gamma _{\rm ac}^{{\rm int}}}$ consisting of available adjacency-configurations;
- integers ${{\rm ac}^{{\rm int}}_{{\rm LB}}}(\gamma ), {{\rm ac}^{{\rm int}}_{{\rm UB}}}(\gamma ) \in [0, {n_{{\rm UB}}}+ |V^{\circ }| - 1], \gamma \in {\Gamma _{\rm ac}^{{\rm int}}}$, that represent the lower and upper bounds of the adjacency-configuration $\gamma$ in $G_{{\mathcal {T}}}$, respectively;

Here, for an adjacency-configuration $\gamma = ({\mathtt a}, {\mathtt b}, m)$, we denote ${{\overline{\gamma }}}:= ({\mathtt b}, {\mathtt a}, m)$. The set ${\Gamma _{\rm ac}^{{\rm int}}}$ is supposed to satisfy $\gamma \in {\Gamma _{\rm ac}^{{\rm int}}}\Rightarrow {{\overline{\gamma }}}\in {\Gamma _{\rm ac}^{{\rm int}}}$.

**Variables**

- Binary variables ${\delta _{\mathrm ac}}(u, [\nu ]; [\gamma ]), u \in V^{\circ }, \nu \in E(C^u), \gamma \in {\Gamma _{\rm ac}^{{\rm int}}}$, indicating whether the edge $\nu$ has adjacency-configuration $\gamma$;
- binary variables ${\delta _{\mathrm ac}}([e]; [\gamma ]), e \in E(T), \gamma \in {\Gamma _{\rm ac}^{{\rm int}}}$, indicating whether the edge *e* has adjacency-configuration $\gamma$;
- integer variables ${{\rm ac}^{{\rm int}}}([\gamma ]) \in [{{\rm ac}^{{\rm int}}_{{\rm LB}}}(\gamma ), {{\rm ac}^{{\rm int}}_{{\rm UB}}}(\gamma )], \gamma \in {\Gamma _{\rm ac}^{{\rm int}}}$, that stores the adjacency-configurations;

**Constraints**

For each $u \in V^{\circ }, \nu = u_iu_j \in E(C^u)$ such that $i < j$:

B67 $$\begin{aligned}&\sum _{\gamma \in {\Gamma _{\rm ac}^{{\rm int}}}} {\delta _{\mathrm ac}}(u, [\nu ]; [\gamma ]) = e_{[\nu ]}^u;&\end{aligned}$$

B68 $$\begin{aligned}&\sum _{\gamma =({\mathtt a},{\mathtt b},m) \in {\Gamma _{\rm ac}^{{\rm int}}}} m \cdot {\delta _{\mathrm ac}}(u, [\nu ]; [\gamma ]) - \beta _{[\nu ]}^u \ge 3(e_{[\nu ]}^u - 1) ;&\nonumber \\&\sum _{\gamma =({\mathtt a},{\mathtt b},m) \in {\Gamma _{\rm ac}^{{\rm int}}}} m \cdot {\delta _{\mathrm ac}}(u, [\nu ]; [\gamma ]) \le \beta _{[\nu ]}^u ;&\end{aligned}$$

B69 $$\begin{aligned}&\sum _{\gamma =({\mathtt a},{\mathtt b},m) \in {\Gamma _{\rm ac}^{{\rm int}}}} [{\mathtt a}] \cdot {\delta _{\mathrm ac}}(u, [\nu ]; [\gamma ]) - \alpha (u,i) \ge |\Lambda | (e_{[\nu ]}^u - 1);&\nonumber \\&\sum _{\gamma =({\mathtt a},{\mathtt b},m) \in {\Gamma _{\rm ac}^{{\rm int}}}} [{\mathtt a}] \cdot {\delta _{\mathrm ac}}(u, [\nu ]; [\gamma ]) \le \alpha (u,i);&\end{aligned}$$

B70 $$\begin{aligned}&\sum _{\gamma =({\mathtt a},{\mathtt b},m) \in {\Gamma _{\rm ac}^{{\rm int}}}} [{\mathtt b}] \cdot {\delta _{\mathrm ac}}(u, [\nu ]; [\gamma ]) - \alpha (u,j) \ge |\Lambda | (e_{[\nu ]}^u - 1);&\nonumber \\&\sum _{\gamma =({\mathtt a},{\mathtt b},m) \in {\Gamma _{\rm ac}^{{\rm int}}}} [{\mathtt b}] \cdot {\delta _{\mathrm ac}}(u, [\nu ]; [\gamma ]) \le \alpha (u,j);&\end{aligned}$$

For each $$e \in E(T)$$:

B71 $$\begin{aligned}&\sum _{\gamma \in {\Gamma _{\rm ac}^{{\rm int}}}} {\delta _{\mathrm ac}}([e]; [\gamma ]) = 1;&\end{aligned}$$

B72 $$\begin{aligned}&\sum _{\gamma =({\mathtt a},{\mathtt b},m) \in {\Gamma _{\rm ac}^{{\rm int}}}} m \cdot {\delta _{\mathrm ac}}([e]; [\gamma ]) = \beta _{[e]};&\end{aligned}$$

For each non-ring edge $e' =uv \in E(T) \setminus E^{\circ }$ such that $[u] < [v]$:

B73 $$\begin{gathered} \sum\limits_{{\gamma = ({\mathtt a},{\mathtt b},m) \in \Gamma _{{\rm ac}}^{{\rm int}} }} {[{\mathtt a}] \cdot \delta _{{{\text{ac}}}} ([e^{\prime}];[\gamma ])} = \alpha (u),\quad {\text{if }}u \notin V^{^\circ } ; \hfill \\ |\Lambda |(x_{{[e^{\prime}],[\mu ]}}^{{{\rm node},u}} - 1) \le \sum\limits_{{\gamma = ({\mathtt a},{\mathtt b},m) \in \Gamma _{{\rm ac}}^{{\rm int}} }} {[{\mathtt a}] \cdot \delta _{{{\text{ac}}}} ([e^{\prime}];[\gamma ])} - \alpha (u,[\mu ]) \le |\Lambda |(1 - x_{{[e^{\prime}],[\mu ]}}^{{{\rm node},u}} ),\quad \mu \in V(C^{u} ),{\text{if }}u \in V^{^\circ } ; \hfill \\ \end{gathered}$$

B74 $$\begin{gathered} \sum\limits_{{\gamma = ({\mathtt a},{\mathtt b},m) \in \Gamma _{{\rm ac}}^{{\rm int}} }} {[{\mathtt b}] \cdot \delta _{{{\text{ac}}}} ([e^{\prime}];[\gamma ])} = \alpha (v),\quad {\text{if }}v \notin V^{\circ } ; \hfill \\ |\Lambda |(x_{{[e^{\prime}],[\mu ]}}^{{{\rm node},v}} - 1) \le \sum\limits_{{\gamma = ({\mathtt a},{\mathtt b},m) \in \Gamma _{{\rm ac}}^{{\rm int}} }} {[{\mathtt b}] \cdot \delta _{{{\text{ac}}}} ([e^{\prime}];[\gamma ])} - \alpha (v,[\mu ]) \le |\Lambda |(1 - x_{{[e^{\prime}],[\mu ]}}^{{{\rm node},v}} ),\quad \mu \in V(C^{v} ),{\text{if }}v \in V^{\circ } ; \hfill \\ \end{gathered}$$

For each $e=uu'\in E^{\circ }$ such that $[u]<[u']$:

B75 $$\begin{aligned} |\Lambda | (x_{[e], [\nu ]}^{{\rm edge}, u} - 1) \le \sum _{\gamma =({\mathtt a}, {\mathtt b}, m) \in {\Gamma _{\rm ac}^{{\rm int}}}} [{\mathtt a}] \cdot {\delta _{\mathrm ac}}([e];[\gamma ]) - \alpha (u, i_1) \le&|\Lambda | (1 - x_{[e], [\nu ]}^{{\rm edge}, u}), \nonumber \\ |\Lambda | (x_{[e], [\nu ]}^{{\rm edge}, u} - 1) \le \sum _{\gamma =({\mathtt a}, {\mathtt b}, m) \in {\Gamma _{\rm ac}^{{\rm int}}}} [{\mathtt b}] \cdot {\delta _{\mathrm ac}}([e];[\gamma ]) - \alpha (u, i_2) \le&|\Lambda | (1 - x_{[e], [\nu ]}^{{\rm edge}, u}), \nonumber \\&\nu = u_{i_1}u_{i_2} \in E(C^u) \ \text {such that } i_1 < i_2; \end{aligned}$$

For each $$\gamma \in {\Gamma _{\rm ac}^{{\rm int}}}$$:

B76 $$\begin{aligned} {\rm{ac}^{\rm{int}}}([\gamma ]) =&\sum _{u \in V^{\circ }} \sum _{\nu \in E(C^u)} ({\delta _{\mathrm ac}}(u, [\nu ];[\gamma ]) + {\delta _{\mathrm ac}}(u, [\nu ]; [{{\overline{\gamma }}}] ) \nonumber \\&+ \sum _{e' \in E(T) \setminus E^{\circ }} ({\delta _{\mathrm ac}}([e'];[\gamma ])+ {\delta _{\mathrm ac}}([e'];[{{\overline{\gamma }}}])) \nonumber \\&- \sum _{e \in E^{\circ }} ({\delta _{\mathrm ac}}([e];[\gamma ]) + {\delta _{\mathrm ac}}([e];[{{\overline{\gamma }}}])),&\text {if } \gamma \ne {{\overline{\gamma }}}; \nonumber \\ {\rm{ac}^{\rm{int}}}([\gamma ]) =&\sum _{u \in V^{\circ }} \sum _{\nu \in E(C^u)} {\delta _{\mathrm ac}}(u, [\nu ];[\gamma ]) + \sum _{e' \in E(T) \setminus E^{\circ }} {\delta _{\mathrm ac}}([e'];[\gamma ]) \nonumber \\&- \sum _{e \in E^{\circ }} {\delta _{\mathrm ac}}([e];[\gamma ]) ,&\text {if } \gamma = {{\overline{\gamma }}}; \end{aligned}$$

### B.8 Descriptors for the number of edge-configurations

**Constants**

- A set ${\Gamma _{\rm ec}^{{\rm int}}}$ consisting of available edge-configurations;
- integers ${\rm{ec}^{{int}}_{\rm{LB}}}(\tau ), {{\rm ec}^{{\rm int}}_{\rm{UB}}}(\tau ) \in [0, {n_{\rm{UB}}}+ |V^{\circ }| - 1], \gamma \in {\Gamma _{\rm ec}^{{\rm int}}}$, that represent the lower and upper bounds of the adjacency-configuration $\tau$ in $G_{{\mathcal {T}}}$, respectively;

Here, for an edge-configuration $\tau = ({\mathtt a}d, {\mathtt b}d', m)$, we denote ${{\overline{\tau }}}:= ({\mathtt b}d', {\mathtt a}d, m)$. The set ${\Gamma _{\rm ec}^{{\rm int}}}$ is supposed to satisfy $\tau \in {\Gamma _{\rm ec}^{{\rm int}}}\Rightarrow {{\overline{\tau }}}\in {\Gamma _{\rm ec}^{{\rm int}}}$.

**Variables**

- Binary variables ${\delta _{\mathrm ec}}(u, [\nu ]; [\tau ]), u \in V^{\circ }, \nu \in E(C^u), \tau \in {\Gamma _{\rm ec}^{{\rm int}}}$, indicating whether the edge $\nu$ has edge-configuration $\tau$;
- binary variables ${\delta _{\mathrm ec}}([e]; [\tau ]), e \in E(T), \tau \in {\Gamma _{\rm ec}^{{\rm int}}}$, indicating whether the edge *e* has edge-configuration $\tau$;
- integer variables ${{\rm ec}^{{\rm int}}}([\tau ]) \in [{{\rm ec}^{{\rm int}}_{\rm{LB}}}(\tau ), {{\rm ec}^{{\rm int}}_{\rm{UB}}}(\tau )], \tau \in {\Gamma _{\rm ec}^{{\rm int}}}$, that stores the edge-configurations;

**Constraints**

For each $u \in V^{\circ }, \nu = u_iu_j \in E(C^u)$ such that $i < j$:

B77 $$\begin{aligned}&\sum _{\tau \in {\Gamma _{\rm ec}^{{\rm int}}}} {\delta _{\mathrm ec}}(u, [\nu ]; [\tau ]) = e_{[\nu ]}^u;&\end{aligned}$$

B78 $$\begin{aligned}&\sum _{\tau = ({\mathtt a}d, {\mathtt b}d', m) \in {\Gamma _{\rm ec}^{{\rm int}}}} [({\mathtt a}, {\mathtt b}, m)] \cdot {\delta _{\mathrm ec}}(u, [\nu ]; [\tau ]) = \sum _{\gamma \in {\Gamma _{\rm ac}^{{\rm int}}}} [\gamma ] \cdot {\delta _{\mathrm ac}}(u, [\nu ];[\gamma ]);&\end{aligned}$$

B79 $$\begin{gathered} \sum\limits_{{\tau = ({\mathtt a}d,{\mathtt b}d^{\prime},m) \in \Gamma _{{\rm ec}}^{{\rm int}} }} d \cdot \delta _{{{\text{ec}}}} (u,[\nu ];[\tau ]) - \sum\limits_{{d \in [1,4]}} d \cdot \delta _{{\rm deg}} (u,i;d) \ge 4(e_{{[\nu ]}}^{u} - 1); \hfill \\ \sum\limits_{{\tau = ({\mathtt a}d,{\mathtt b}d^{\prime},m) \in \Gamma _{{\rm ec}} ^{{\rm int}} }} d \cdot \delta _{{{\text{ec}}}} (u,[\nu ];[\tau ]) \le \sum\limits_{{d \in [1,4]}} d \cdot \delta _{{\rm deg}} (u,i;d); \hfill \\ \end{gathered}$$

B80 $$\begin{gathered} \sum\limits_{{\tau = ({\mathtt a}d,{\mathtt b}d^{\prime},m) \in \Gamma _{{\rm ec}}^{{\rm int}} }} {d^{\prime}} \cdot \delta _{{{\text{ec}}}} (u,[\nu ];[\tau ]) - \sum\limits_{{d^{\prime} \in [1,4]}} {d^{\prime}} \cdot \delta _{{\rm deg}} (u,j;d^{\prime}) \ge 4(e_{{[\nu ]}}^{u} - 1); \hfill \\ \sum\limits_{{\tau = ({\mathtt a}d,{\mathtt b}d^{\prime},m) \in \Gamma _{{\rm ec}}^{{\rm int}} }} {d^{\prime}} \cdot \delta _{{{\text{ec}}}} (u,[\nu ];[\tau ]) \le \sum\limits_{{d^{\prime} \in [1,4]}} {d^{\prime}} \cdot \delta _{{\rm deg}} (u,j;d^{\prime}); \hfill \\ \end{gathered}$$

For each $$e \in E(T)$$:

B81 $$\begin{aligned}&\sum _{\tau \in {\Gamma _{\rm ec}^{{\rm int}}}} {\delta _{\mathrm ec}}([e];[\tau ]) = 1;&\end{aligned}$$

B82 $$\begin{aligned}&\sum _{\tau = ({\mathtt a}d, {\mathtt b}d', m) \in {\Gamma _{\rm ec}^{{\rm int}}}} [({\mathtt a}, {\mathtt b}, m)] \cdot {\delta _{\mathrm ec}}([e]; [\tau ]) = \sum _{\gamma \in {\Gamma _{\rm ac}^{{\rm int}}}} [\gamma ] \cdot {\delta _{\mathrm ac}}([e]; [\gamma ]);&\end{aligned}$$

For each non-ring edge $e' =uv \in E(T) \setminus E^{\circ }$ such that $[u] < [v]$:

B83 $$\begin{gathered} \sum\limits_{{\tau = ({\mathtt a}d,{\mathtt b}d^{\prime},m) \in \Gamma _{{\rm ec}}^{{{\text{int}}}} }} d \cdot \delta _{{{\text{ec}}}} ([e^{\prime}];[\tau ]) = \sum\limits_{{d \in [1,4]}} d \cdot \delta _{{\rm deg}} (u;d),\quad {\text{if }}u \notin V^{^\circ } ; \hfill \\ \sum\limits_{{\tau = ({\mathtt a}d,{\mathtt b}d^{\prime},m) \in \Gamma _{{\rm ec}} ^{{\rm int}} }} d \cdot \delta _{{{\text{ec}}}} ([e^{\prime}];[\tau ]) - \sum\limits_{{d \in [1,4]}} d \cdot \delta _{{\rm deg}} (u,[\mu ];d) \ge 4(x_{{[e^{\prime}],[\mu ]}}^{{{\rm node},u}} - 1), \hfill \\ \sum\limits_{{\tau = ({\mathtt a}d,{\mathtt b}d^{\prime},m) \in \Gamma _{{\rm ec}}^{{\rm int}} }} d \cdot \delta _{{{\text{ec}}}} ([e^{\prime}];[\tau ]) - \sum\limits_{{d \in [1,4]}} d \cdot \delta _{{\rm deg}} (u,[\mu ];d) \le 4(1 - x_{{[e^{\prime}],[\mu ]}}^{{{\rm node},u}} ),\quad \mu \in V(C^{u} ),\,\,{\text{if }}u \in V^{\circ } ; \hfill \\ \end{gathered}$$

B84 $$\begin{gathered} \sum\limits_{{\tau = ({\mathtt a}d,{\mathtt b}d^{\prime},m) \in \Gamma _{{\rm ec}}^{{\rm int}} }} {d^{\prime}} \cdot \delta _{{{\text{ac}}}} ([e^{\prime}];[\tau ]) = \sum\limits_{{d^{\prime} \in [1,4]}} {d^{\prime}} \cdot \delta _{{\rm deg}} (v;d),\quad {\text{if }}v \notin V^{\circ } ; \hfill \\ \sum\limits_{{\tau = ({\mathtt a}d,{\mathtt b}d^{\prime},m) \in \Gamma _{{\rm ec}}^{{\rm int}} }} {d^{\prime}} \cdot \delta _{{{\text{ec}}}} ([e^{\prime}];[\tau ]) - \sum\limits_{{d^{\prime} \in [1,4]}} {d^{\prime}} \cdot \delta _{{\rm deg}} (v,[\mu ];d^{\prime}) \ge 4(x_{{[e^{\prime}],[\mu ]}}^{{{\rm node},v}} - 1), \hfill \\ \sum\limits_{{\tau = ({\mathtt a}d,{\mathtt b}d^{\prime},m) \in \Gamma _{{\rm ec}}^{{{\text{int}}}} }} {d^{\prime}} \cdot \delta _{{{\text{ec}}}} ([e^{\prime}];[\tau ]) - \sum\limits_{{d^{\prime} \in [1,4]}} {d^{\prime}} \cdot \delta _{{\rm deg}} (v,[\mu ];d^{\prime}) \le 4(1 - x_{{[e^{\prime}],[\mu ]}}^{{{\rm node},v}} ),\quad \mu \in V(C^{v} ),{\text{if }}v \in V^{\circ } ; \hfill \\ \end{gathered}$$

For each $e=uu'\in E^{\circ }$ such that $[u]<[u']$:

B85 $$\begin{aligned} 4 (x_{[e], [\nu ]}^{{\rm edge}, u} - 1) \le \sum _{\tau =({\mathtt a}d, {\mathtt b}d', m) \in {\Gamma _{\rm ec}^{{\rm int}}}} d \cdot {\delta _{\mathrm ec}}([e];[\tau ])&- \sum _{d \in [1,4]} d \cdot {\delta _{{\rm deg}}}(u, i_1; d) \le 4(1 - x_{[e], [\nu ]}^{{\rm edge}, u}), \nonumber \\ 4 (x_{[e], [\nu ]}^{{\rm edge}, u} - 1) \le \sum _{\tau =({\mathtt a}d, {\mathtt b}d', m) \in {\Gamma _{\rm ec}^{{\rm int}}}} d' \cdot {\delta _{\mathrm ec}}([e];[\tau ])&- \sum _{d' \in [1,4]} d' \cdot {\delta _{{\rm deg}}}(u, i_2; d) \le 4 (1 - x_{[e], [\nu ]}^{{\rm edge}, u}), \nonumber \\&\nu = u_{i_1}u_{i_2} \in E(C^u) \ \text {such that } i_1 < i_2; \end{aligned}$$

For each $$\tau \in {\Gamma _{\rm ec}^{{\rm int}}}$$:

B86 $$\begin{aligned} {\rm{ec}^{\rm{int}}}([\tau ]) =&\sum _{u \in V^{\circ }} \sum _{\mu \in V(C^u)} ({\delta _{\mathrm ec}}(u, [\mu ];[\tau ]) + {\delta _{\mathrm ec}}(u, [\mu ]; [{{\overline{\tau }}}] ) \nonumber \\&+ \sum _{e' \in E(T) \setminus E^{\circ }} ({\delta _{\mathrm ec}}([e'];[\tau ])+ {\delta _{\mathrm ec}}([e'];[{{\overline{\tau }}}])) \nonumber \\&- \sum _{e \in E^{\circ }} ({\delta _{\mathrm ec}}([e];[\tau ]) + {\delta _{\mathrm ec}}([e];[{{\overline{\tau }}}])),&\text {if } \tau \ne {{\overline{\tau }}}; \nonumber \\ {\rm{ec}^{\rm{int}}}([\tau ]) =&\sum _{u \in V^{\circ }} \sum _{\mu \in V(C^u)} {\delta _{\mathrm ec}}(u, [\mu ];[\tau ]) + \sum _{e' \in E(T) \setminus E^{\circ }} {\delta _{\mathrm ec}}([e'];[\tau ]) \nonumber \\&- \sum _{e \in E^{\circ }} {\delta _{\mathrm ec}}([e];[\tau ]) ,&\text {if } \tau = {{\overline{\tau }}}; \end{aligned}$$

## References

[1] Cherkasov A, Muratov EN, Fourches D, Varnek A, Baskin II, Cronin M, Dearden J, Gramatica P, Martin YC, Todeschini R, Consonni V, Kuz’min VE, Cramer R, Benigni R, Yang C, Rathman J, Terfloth L, Gasteiger J, Richard A, Tropsha A (2014) Qsar modeling: Where have you been? where are you going to? J Med Chem 57(12):4977–5010. 10.1021/jm4004285 24351051 10.1021/jm4004285 PMC4074254

[2] Tropsha A, Isayev O, Varnek A, Schneider G, Cherkasov A (2024) Integrating QSAR modelling and deep learning in drug discovery: the emergence of deep QSAR. Nat Rev Drug Disc 23(2):141–155. 10.1038/s41573-023-00832-0 10.1038/s41573-023-00832-0 38066301

[3] Ikebata H, Hongo K, Isomura T, Maezono R, Yoshida R (2017) Bayesian molecular design with a chemical language model. J Comput Aided Mol Des 31:379–391. 10.1007/s10822-016-0008-z 28281211 10.1007/s10822-016-0008-z PMC5393296

[4] Rupakheti C, Virshup A, Yang W, Beratan DN (2015) Strategy to discover diverse optimal molecules in the small molecule universe. J Chem Inf Model 55(3):529–537. 10.1021/ci500749q 25594586 10.1021/ci500749q PMC4372820

[5] Miyao T, Kaneko H, Funatsu K (2016) Inverse QSPR/QSAR analysis for chemical structure generation (from y to x). J Chem Inf Model 56(2):286–299. 10.1021/acs.jcim.5b00628 26818135 10.1021/acs.jcim.5b00628

[6] Kaneko H (2023) Molecular descriptors, structure generation, and inverse QSAR/QSPR based on SELFIES. ACS Omega 8(24):21781–21786. 10.1021/acsomega.3c01332 37360490 10.1021/acsomega.3c01332 PMC10286088

[7] Zdrazil B, Felix E, Hunter F, Manners EJ, Blackshaw J, Corbett S, Veij M, Ioannidis H, Lopez DM, Mosquera JF, Magarinos MP, Bosc N, Arcila R, Kizilören T, Gaulton A, Bento AP, Adasme MF, Monecke P, Landrum GA, Leach AR (2023) The ChEMBL database in 2023: a drug discovery platform spanning multiple bioactivity data types and time periods. Nucl Acids Res 52(D1):1180–1192. 10.1093/nar/gkad1004 10.1093/nar/gkad1004 PMC10767899 37933841

[8] Morgan D, Jacobs R (2020) Opportunities and challenges for machine learning in materials science. Annu Rev Mater Res 50:71–103. 10.1146/annurev-matsci-070218-010015

[9] Ghasemi F, Mehridehnavi A, Pérez-Garrido A, Pérez-Sánchez H (2018) Neural network and deep-learning algorithms used in QSAR studies: merits and drawbacks. Drug Disc Today 23:1784–1790. 10.1016/j.drudis.2018.06.016 10.1016/j.drudis.2018.06.016 29936244

[10] Bilodeau C, Jin W, Jaakkola T, Barzilay R, Jensen KF (2022) Generative models for molecular discovery: recent advances and challenges. WIREs Computat Mol Sci 12(5):1608. 10.1002/wcms.1608

[11] Gómez-Bombarelli R, Wei JN, Duvenaud D, Hernández-Lobato JM, Sánchez-Lengeling B, Sheberla D, Aguilera-Iparraguirre J, Hirzel TD, Adams RP, Aspuru-Guzik A (2018) Automatic chemical design using a data-driven continuous representation of molecules. ACS Cent Sci 4(2):268–276. 10.1021/acscentsci.7b00572 29532027 10.1021/acscentsci.7b00572 PMC5833007

[12] Kwon K, Jeong K, Park J, Na H, Shin J (2023) String-based molecule generation via multi-decoder VAE. In: ICASSP 2023 - 2023 IEEE International Conference on Acoustics, Speech and Signal Processing (ICASSP), pp. 1–5. 10.1109/ICASSP49357.2023.10095212

[13] Ma C, Zhang X (2021) GF-VAE: A flow-based variational autoencoder for molecule generation. In: Proceedings of the 30th ACM International Conference on Information & Knowledge Management. CIKM ’21, pp. 1181–1190. Association for Computing Machinery, New York, NY, USA. 10.1145/3459637.3482260

[14] Wu H, Ye X, Yan J (2024) QVAE-mole: The quantum VAE with spherical latent variable learning for 3-D molecule generation. In: The Thirty-eighth Annual Conference on Neural Information Processing Systems. https://openreview.net/forum?id=RqvesBxqDo

[15] Cao ND, Kipf T (2022) MolGAN: An implicit generative model for small molecular graphs. https://arxiv.org/abs/1805.11973

[16] Prykhodko O, Johansson SV, Kotsias P-C, Arús-Pous J, Bjerrum EJ, Engkvist O, Chen H (2019) A de novo molecular generation method using latent vector based generative adversarial network. J Chem 11(1):74. 10.1186/s13321-019-0397-9 10.1186/s13321-019-0397-9 PMC6892210 33430938

[17] Li C, Yamanishi Y (2024) TenGAN: Pure transformer encoders make an efficient discrete GAN for de novo molecular generation. In: Dasgupta, S., Mandt, S., Li, Y. (eds.) Proceedings of The 27th International Conference on Artificial Intelligence and Statistics. Proceedings of Machine Learning Research, vol. 238, pp. 361–369. PMLR, Palau de Congressos, Valencia, Spain. https://proceedings.mlr.press/v238/li24d.html

[18] Madhawa K, Ishiguro K, Nakago K, Abe M (2019) GraphNVP: An Invertible Flow Model for Generating Molecular Graphs. arxiv:org/abs/1905.11600

[19] Shi C, Xu M, Zhu Z, Zhang W, Zhang M, Tang J (2020) GraphAF: a Flow-based Autoregressive Model for Molecular Graph Generation. arXiv:abs/2001.09382

[20] Ma C, Yang Q, Gao X, Zhang X (2022) DEMO: Disentangled molecular graph generation via an invertible flow model. In: Proceedings of the 31st ACM International Conference on Information & Knowledge Management. CIKM ’22, pp. 1420–1429. Association for Computing Machinery, New York, NY, USA. 10.1145/3511808.3557217

[21] Hua C, Luan S, Xu M, Ying Z, Fu J, Ermon S, Precup D (2024) MUDiff: Unified diffusion for complete molecule generation. In: Villar, S., Chamberlain, B. (eds.) Proceedings of the Second Learning on Graphs Conference. Proceedings of Machine Learning Research, vol 231, pp 33–13326. PMLR, Virtual . https://proceedings.mlr.press/v231/hua24a.html

[22] Xu M, Powers AS, Dror RO, Ermon S, Leskovec J (2023) Geometric latent diffusion models for 3D molecule generation. In: Krause, A., Brunskill, E., Cho, K., Engelhardt, B., Sabato, S., Scarlett, J. (eds.) Proceedings of the 40th International Conference on Machine Learning. Proceedings of Machine Learning Research, vol. 202, pp. 38592–38610. PMLR, Honolulu, Hawaii, USA. https://proceedings.mlr.press/v202/xu23n.html

[23] Bagal V, Aggarwal R, Vinod PK, Priyakumar UD (2022) MolGPT: Molecular generation using a transformer-decoder model. J Chem Inf Model 62(9):2064–2076. 10.1021/acs.jcim.1c00600 34694798 10.1021/acs.jcim.1c00600

[24] Grechishnikova D (2021) Transformer neural network for protein-specific de novo drug generation as a machine translation problem. Sci Rep 11(1):321. 10.1038/s41598-020-79682-4 33432013 10.1038/s41598-020-79682-4 PMC7801439

[25] Ross J, Belgodere B, Hoffman SC, Chenthamarakshan V, Navratil J, Mroueh Y, Das P (2025) GP-MoLFormer: A Foundation Model For Molecular Generation. arXiv:org/abs/2405.04912

[26] Mazuz E, Shtar G, Shapira B, Rokach L (2023) Molecule generation using transformers and policy gradient reinforcement learning. Sci Rep 13(1):8799. 10.1038/s41598-023-35648-w 37258546 10.1038/s41598-023-35648-w PMC10232454

[27] Zhu J (2023) Novel methods for chemical compound inference based on machine learning and mixed integer linear programming. PhD thesis, Kyoto University. http://hdl.handle.net/2433/285872

[28] Akutsu T, Nagamochi H (2019) A mixed integer linear programming formulation to artificial neural networks. Technical Report 2019-001, Department of Applied Mathematics and Physics, Graduate School of Informatics, Kyoto University. http://www.amp.i.kyoto-u.ac.jp/tecrep/ps_file/2019/2019-001.pdf

[29] Zhu J, Azam NA, Haraguchi K, Zhao L, Nagamochi H, Akutsu T (2022) An inverse QSAR method based on linear regression and integer programming. Frontiers in Bioscience-Landmark **27**(6), 188 10.31083/j.fbl2706188 . The preprint appears as arXiv:2107.02381 10.31083/j.fbl2706188 35748264

[30] Tanaka K, Zhu J, Azam NA, Haraguchi K, Zhao L, Nagamochi H, Akutsu T (2021) An inverse QSAR method based on decision tree and integer programming. In: Proceedings of The 17th International Conference on Intelligent Computing, Lecture Notes in Computer Science, Vol. 12837, August in Shenzhen, China, pp. 628–644. 10.1007/978-3-030-84529-2_53

[31] Azam NA, Chiewvanichakorn R, Zhang F, Shurbevski A, Nagamochi H, Akutsu T (2020) A method for the inverse QSAR/QSPR based on artificial neural networks and mixed integer linear programming. In: Proceedings of the 13th International Joint Conference on Biomedical Engineering Systems and Technologies – Volume 3: BIOINFORMATICS, pp. 101–108. 10.1145/3386052.3386054

[32] Zhang F, Zhu J, Chiewvanichakorn R, Shurbevski A, Nagamochi H, Akutsu T (2022) A new approach to the design of acyclic chemical compounds using skeleton trees and integer linear programming. Appl Intell 52(15):17058–17072. 10.1007/s10489-021-03088-6

[33] Ito R, Azam NA, Wang C, Shurbevski A, Nagamochi H, Akutsu T (2021) A novel method for the inverse qsar/qspr to monocyclic chemical compounds based on artificial neural networks and integer programming. In: Arabnia HR, Deligiannidis L, Shouno H, Tinetti FG, Tran Q-N (eds) Advances in Computer Vision and Computational Biology. Springer, Cham

[34] Zhu J, Wang C, Shurbevski A, Nagamochi H, Akutsu T (2020) A novel method for inference of chemical compounds of cycle index two with desired properties based on artificial neural networks and integer programming. Algorithms 13:124. 10.3390/a13050124 10.1186/s13015-021-00197-2 PMC8364129 34391471

[35] Shi Y, Zhu J, Azam NA, Haraguchi K, Zhao L, Nagamochi H, Akutsu T (2021) An inverse QSAR method based on a two-layered model and integer programming. Int J Mol Sci 22:2847. 10.3390/ijms22062847 33799613 10.3390/ijms22062847 PMC8002091

[36] Ido R, Cao S, Zhu J, Azam NA, Haraguchi K, Zhao L, Nagamochi H, Akutsu T (2024) A method for inferring polymers based on linear regression and integer programming. IEEE/ACM Trans Comput Biol Bioinf 21(6):1623–1632. 10.1109/TCBB.2024.3447780 10.1109/TCBB.2024.3447780 39172611

[37] Song B, Zhu J, Azam NA, Haraguchi K, Zhao L, Akutsu T (2024) Cycle-Configuration: A Novel Graph-theoretic Descriptor Set for Molecular Inference. arXiv:abs/2408.05136 10.1186/s13321-025-01042-z PMC12360023 40826377

[38] Song B, Zhu J, Azam NA, Haraguchi K, Zhao L, Akutsu T (2024) Cycle-configuration: A novel graph-theoretic descriptor set for molecular inference. In: 2024 IEEE International Conference on Bioinformatics and Biomedicine (BIBM), p 856–859. 10.1109/BIBM62325.2024.10822617

[39] Tox21 Data Challenge 2014. https://tripod.nih.gov/tox21/challenge/data.jsp, accessed on February 5th, 2025

[40] MoleculeNet. http://moleculenet.org, accessed on February 5th, 2025

[41] Annotations from HSDB (on PubChem). https://pubchem.ncbi.nlm.nih.gov/, accessed on February 5th, 2025

[42] Jalali-Heravi M, Fatemi MH (2001) Artificial neural network modeling of kováts retention indices for noncyclic and monocyclic terpenes. J Chromatogr A 915(1):177–183. 10.1016/S0021-9673(00)01274-7 11358247 10.1016/s0021-9673(00)01274-7

[43] Naef R (2019) Calculation of the isobaric heat capacities of the liquid and solid phase of organic compounds at and a round 29815 k based on their true molecular volume. Molecules. 10.3390/molecules24081626 31022983 10.3390/molecules24081626 PMC6514989

[44] Goussard V, Duprat F, Gerbaud V, Ploix J-L, Dreyfus G, Nardello-Rataj V, Aubry J-M (2017) Predicting the surface tension of liquids: Comparison of four modeling approaches and application to cosmetic oils. J Chem Inf Model 57(12):2986–2995. 10.1021/acs.jcim.7b00512 29091426 10.1021/acs.jcim.7b00512

[45] Goussard V, Duprat F, Ploix J-L, Dreyfus G, Nardello-Rataj V, Aubry J-M (2020) A new machine-learning tool for fast estimation of liquid viscosity application to cosmetic oils. J Chem Inform Model 60(4):2012–2023. 10.1021/acs.jcim.0c00083 10.1021/acs.jcim.0c00083 32250628

[46] Debnath AK, Compadre RL, Debnath G, Shusterman AJ, Hansch C (1991) Structure-activity relationship of mutagenic aromatic and heteroaromatic nitro compounds correlation with molecular orbital energies and hydrophobicity. J Med Chem 34(2):786–797. 10.1021/jm00106a046 1995902 10.1021/jm00106a046

[47] Helma C, King RD, Kramer S, Srinivasan A (2001) The predictive toxicology challenge 2000–2001. Bioinformatics 17(1):107–108. 10.1093/bioinformatics/17.1.107

[48] Tibshirani R (1996) Regression shrinkage and selection via the lasso. J Roy Stat Soc: Ser B 58:267–288. 10.1111/j.2517-6161.1996.tb02080.x

[49] Quinlan JR (1986) Induction of decision trees. Mach Learn 1:81–106. 10.1007/BF00116251

[50] Rumelhart DE, Hinton GE, Williams RJ (1986) Learning representations by back-propagating errors. Nature 323(6088):533–536. 10.1038/323533a0

[51] Breiman L (2001) Random forests. Mach Learn 45:5–32. 10.1023/A:1010933404324

[52] IBM ILOG CPLEX Optimization Studio. https://www.ibm.com/products/ilog-cplex-optimization-studio, accessed on February 5th, 2025

[53] Cheng Y, Gong Y, Liu Y, Song B, Zou Q (2021) Molecular design in drug discovery: a comprehensive review of deep generative models. Brief Bioinform 22(6):344. 10.1093/bib/bbab344 10.1093/bib/bbab344 34415297

[54] Zhu J, Azam NA, Zhang F, Shurbevski A, Haraguchi K, Zhao L, Nagamochi H, Akutsu T (2022) A novel method for inferring chemical compounds with prescribed topological substructures based on integer programming. IEEE/ACM Trans Comput Biol Bioinf 19(6):3233–3245. 10.1109/TCBB.2021.3112598 10.1109/TCBB.2021.3112598 34520360

[55] Azam N, Zhu J, Haraguchi K, Zhao L, Nagamochi H, Akutsu T (2021) Molecular design based on artificial neural networks, integer programming and grid neighbor search. In: 2021 IEEE International Conference on Bioinformatics and Biomedicine (BIBM), pp. 360–363. IEEE Computer Society, Los Alamitos, CA, USA. 10.1109/BIBM52615.2021.9669710

[56] Azam NA, Zhu J, Sun Y, Shi Y, Shurbevski A, Zhao L, Nagamochi H, Akutsu T (2021) A novel method for inference of acyclic chemical compounds with bounded branch-height based on artificial neural networks and integer programming. Algor Mol Biol 16:1–39. 10.1186/s13015-021-00197-2 10.1186/s13015-021-00197-2 PMC8364129 34391471

